# Habitat geography around Hawaii’s oceanic islands influences tiger shark (*Galeocerdo cuvier*) spatial behaviour and shark bite risk at ocean recreation sites

**DOI:** 10.1038/s41598-018-23006-0

**Published:** 2018-03-21

**Authors:** Carl G. Meyer, James M. Anderson, Daniel M. Coffey, Melanie R. Hutchinson, Mark A. Royer, Kim N. Holland

**Affiliations:** 1 Hawaii Institute of Marine Biology, University of Hawaii at Manoa, P. O. Box, 1346 Kaneohe, Hawaii USA; 2 Joint Institute for Marine and Atmospheric Research, Pacific Islands Fisheries Science Center, 1845 Wasp Blvd. Bldg. 176, Honolulu, Hawaii 96818 USA

## Abstract

We compared tiger shark (*Galeocerdo cuvier*) spatial behaviour among 4 Hawaiian Islands to evaluate whether local patterns of movement could explain higher numbers of shark bites seen around Maui than other islands. Our sample consisted of 96 electronically-tagged (satellite and acoustic transmitters) tiger sharks, individually tracked for up to 6 years. Most individuals showed fidelity to a specific ‘home’ island, but also swam between islands and sometimes ranged far (up to 1,400 km) offshore. Movements were primarily oriented to insular shelf habitat (0–200 m depth) in coastal waters, and individual sharks utilized core-structured home ranges within this habitat. Core utilization areas of large tiger sharks were closer to high-use ocean recreation sites around Maui, than around Oahu. Tiger sharks routinely visited shallow ocean recreation sites around Maui and were detected on more days overall at ocean recreation sites around Maui (62–80%) than Oahu (<6%). Overall, our results suggest the extensive insular shelf surrounding Maui supports a fairly resident population of tiger sharks and also attracts visiting tiger sharks from elsewhere in Hawaii. Collectively these natural, habitat-driven spatial patterns may in-part explain why Maui has historically had more shark bites than other Hawaiian Islands.

## Introduction

Over the past 20 years, the island of Maui has experienced twice as many shark bite incidents as Oahu despite the fact that the human population of Oahu is six times larger than that of Maui (Supplementary Table S1; Supplementary Figs S1 and S2). Obvious questions to ask are whether sharks are more abundant and/or have greater overlap with recreational water use around Maui compared to Oahu, and other Hawaiian Islands, where fewer shark bites have occurred? Specifically, do sharks around Maui show any evidence of being more resident (“site-attached”), visiting coastal recreation sites more often, or spending more time in these areas, than sharks captured around other Hawaiian Islands? Similarly, are there any seasonal or episodic influxes of sharks into Maui waters from other areas?

Tiger sharks (*Galeocerdo cuvier*) are the most commonly identified species in Hawaii shark bite incidents^1^, and based on their large size, broad diet and local abundance, are thought to be responsible for most of the remaining bites where the species was not identified^1^. Although tiger shark movements around Maui have received scant previous attention, their movements have been extensively studied around several other Hawaiian Islands (e.g. Oahu^2^, Hawaii Island^3^, French Frigate Shoals [FFS]^4,5^). These previous studies provide baseline behaviour patterns for comparison with Maui sharks. Moreover, these studies demonstrated that simultaneous use of satellite and acoustic telemetry (i.e. equipping individual sharks with two different types of transmitter) provides a detailed overview of tiger shark movements, and can reveal complex dispersal patterns potentially linked to both foraging and breeding. Individual tiger sharks tend to utilize a particular ‘core’ island, but also swim between islands and range extensively offshore^2–5^. Models predict that 25% of mature females swim from FFS to the Main Hawaiian Islands (MHI) during late summer/early fall, potentially to give birth (individual females give birth every third year^6^). Females with core home ranges within the MHI remain within this region, where movements between islands are better explained by sea surface temperature and chlorophyll *a* concentration, suggesting they may be driven by foraging^5^.

In this study we used satellite and acoustic telemetry to quantify the movements of tiger sharks captured off high use ocean recreation sites around Maui and Oahu. Our broad goal was to determine whether the higher number of shark bites occurring around Maui could be reasonably explained by differences in tiger shark movements and habitat use between Maui and other Hawaiian islands. Specifically we evaluated (1) whether tiger sharks captured around Maui visited human ocean recreation sites more often, or for longer periods, than tiger sharks captured around other islands, (2) whether Maui waters experience seasonal or episodic influxes of sharks from other areas, and (3) whether tiger shark movements and home range characteristics are linked to habitat bathymetry.

## Results

### Overview

From 2013–2015, a total of 41 tiger sharks (217 to 448 cm Total Length [TL]) were captured around Maui (26 sharks) and Oahu (15 sharks), and equipped with electronic tags (Supplementary Table S2). Of these, 28 sexually mature individuals were equipped with both surgically-implanted acoustic tags and dorsal fin-mounted satellite transmitters, and the remaining 13 with acoustic tags only (Supplementary Table S2). Thirty-eight (93%) of these sharks were subsequently detected via either satellite or acoustic transmissions over periods ranging from 7–613 days (Supplementary Table 2). Two sharks were also equipped with video cameras, with useable footage recovered from one individual (the camera detached prematurely from the other shark)(Supplementary Table 2).

In addition, 20 “legacy” tiger sharks acoustically-tagged at 4 Hawaiian Islands (but primarily around Oahu) prior to this study, were theoretically still detectable (because their transmitters were still active) during the time frame of the current study (Supplementary Table S3). Five (25%) of these sharks were detected around Maui between October 2013 and June 2015. All 5 of these legacy sharks were originally captured and tagged around Oahu, with the earliest tagging event for a detected legacy shark occurring in February 2009 (Supplementary Table S3).

For comparative purposes, we also included historical data from tiger sharks acoustically-tagged around Hawaii Island (Supplementary Table S4) and at FFS (Supplementary Table S5) in our analyses of tiger shark spatial dynamics. Hawaii Island tiger shark data were obtained from 11 individuals ranging in size from 181 to 460 cm TL, and detected over periods ranging from <1–924 days (Supplementary Table S4). FFS data were derived from 39 tiger sharks ranging in size from 261 to 450 cm TL, and detected over periods ranging from <1 to 949 days (Supplementary Table 5). A non-parametric Kruskal-Wallis test revealed no significant differences in mean tiger shark size among tagging islands (n = 90, df = 3, H = 7.722, P = 0.0521).

### Dorsal fin-mounted satellite tags

Twenty-five (89%) satellite-tagged tiger sharks yielded Argos location estimates over periods ranging from 7 to 595 days (median 164 days), and were detectable for up to 756 days (Supplementary Table S2). In the latter case, the unique satellite-tag identification codes continued to be registered by both satellite and land-based receivers^7^, but data were insufficient for calculating Argos location estimates, although detection via the land-based receivers indicated these sharks were still present in waters around Maui.

**Figure 1:**
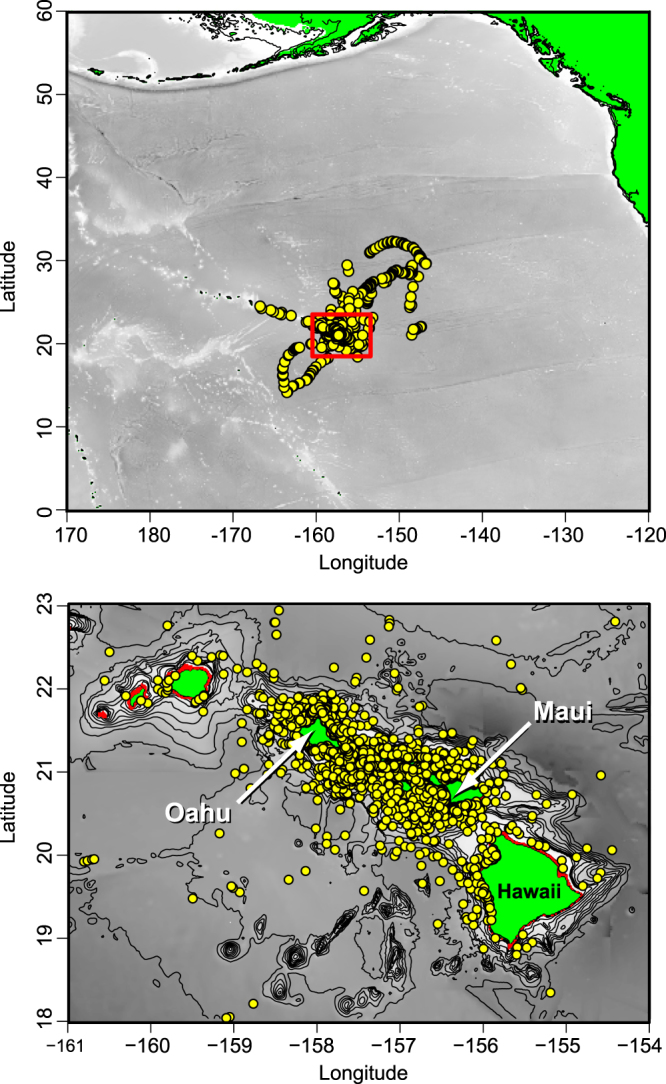
Location estimates for tiger sharks equipped with dorsal fin-mounted satellite transmitters (SPOT tags). Top panel: Overview of filtered Argos locations (yellow points) from 32 tiger sharks captured off Maui and Oahu. Red box indicates area shown in detail in bottom panel. Bottom panel: Tiger shark filtered Argos locations (yellow points) within coastal habitats of the Main Hawaiian Islands. Maps were created using R software v.3.1.2 (R: A Language and Environment for Statistical Computing, R Core Team, R Foundation for Statistical Computing, Vienna, Austria (2014) https://www.R-project.org).

**Figure 2:**
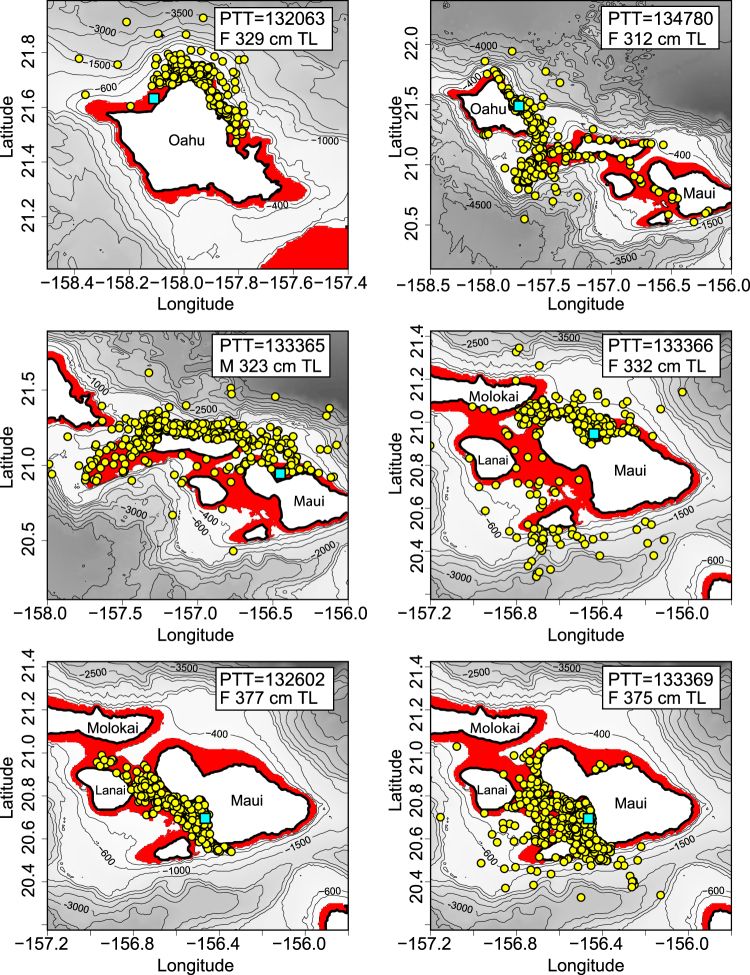
Filtered Argos locations (yellow points) of six individual tiger sharks (M/F = sex, TL = Total Length in cm) showing concentration over insular shelf habitat (0–200 m depth, red shading). Light blue squares indicate tagging locations of each shark (Two were tagged off Oahu, four off Maui). Maps were created using R software v.3.1.2 (R: A Language and Environment for Statistical Computing, R Core Team, R Foundation for Statistical Computing, Vienna, Austria (2014) https://www.R-project.org).

Satellite tracking data revealed extensive (>100 km up to 1,460 km) offshore movements by seven tiger sharks, with some individuals spending months in open-ocean with only brief visits to coastal waters, but the majority (78.7%) of satellite location estimates occurred in coastal (<500 m depth) waters around Oahu, Maui and Hawaii Island (Figs 1 and 2). Most individuals were detected most frequently in waters around their tagging island, but sharks tagged around Maui also visited waters around Molokai, Lanai, Kahoolawe and Hawaii Island. Tiger sharks tagged around Oahu also visited waters around Kauai, Molokai, Maui and Lanai.

#### Coastal home range analyses of satellite tracking data

**Figure 3:**
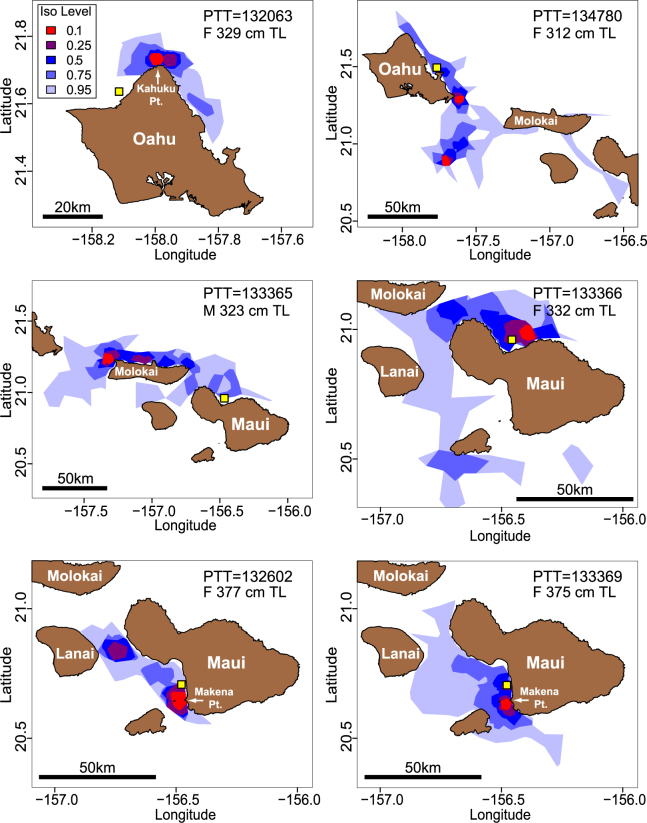
Home range isopleths for tiger sharks captured around Maui and Oahu. Yellow square indicates original tagging location. Isopleth levels indicate the proportion of total satellite locations enclosed by the polygon (e.g. area in red = 10%, dark blue = 50%, light blue = 95%). Maps were created using R software v.3.1.2 (R: A Language and Environment for Statistical Computing, R Core Team, R Foundation for Statistical Computing, Vienna, Austria (2014) https://www.R-project.org).

**Figure 4:**
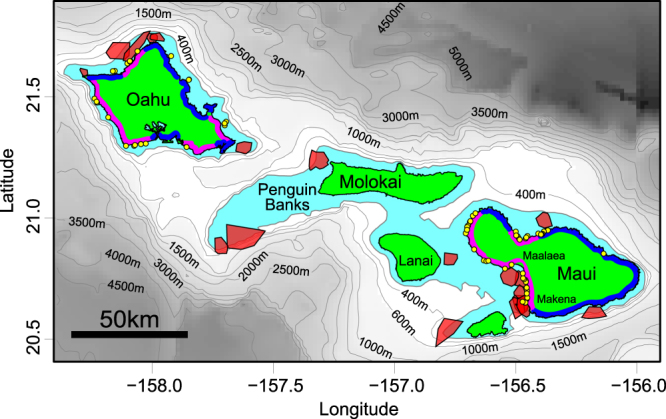
Home range core use areas (0.1 iso level - translucent red polygons) of 19 satellite-tagged tiger sharks captured around Maui (n = 13) and Oahu (n = 6). Note the cluster of core areas in waters off SW Maui (overlapping core areas of 7 individuals were identified in waters between Maalaea and Makena). Light-blue shaded area indicates insular shelf (depth 0–200 m). Yellow points indicate locations of documented shark bite incidents along high recreational-use (pink) and low recreational-use (dark-blue) coastlines. Maps were created using R software v.3.1.2 (R: A Language and Environment for Statistical Computing, R Core Team, R Foundation for Statistical Computing, Vienna, Austria (2014) https://www.R-project.org).

Satellite tracking revealed that each shark had unique home range characteristics but there were several common themes of space and habitat use among individuals. For example, tiger shark home ranges typically included waters around several adjacent islands, and sharks were most frequently detected over insular shelf habitat within the 200-m isobath (Fig. 1). Nineteen satellite-tagged sharks yielded sufficient positions for home range isopleth analyses (Figs 3 and 4). Most of these individuals utilized clearly-defined core areas (the area where satellite fixes were most strongly clustered) associated with relatively wide areas of insular shelf (Figs 3 and 4). Tiger shark core use areas around Maui were often adjacent to ocean recreation beaches, with overlapping core areas of 7 tagged individuals identified in waters off SW Maui (Fig. 4). Around Oahu, overlapping tiger shark home range cores were documented in waters off the North coast, and some Oahu-tagged tiger sharks also utilized core use areas on Penguin Banks (a westward extension of the insular shelf surrounding Maui County - Fig. 4).

#### Bathymetry profiles from Argos locations

**Figure 5:**
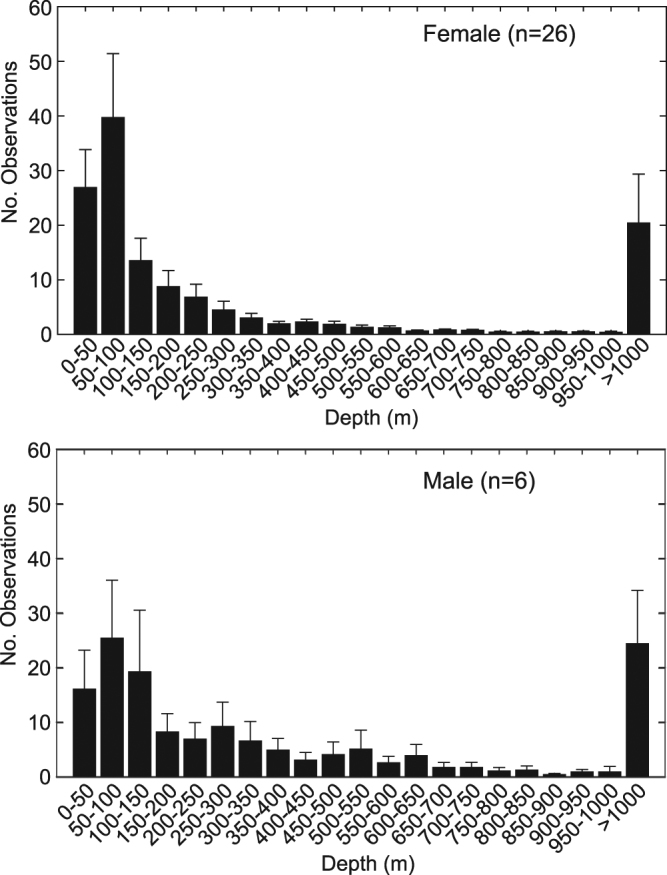
Depth frequency distribution (mean ± standard error) of bathymetry corresponding with Argos locations of female (top panel) and male (bottom panel) tiger sharks equipped with dorsal fin-mounted satellite transmitters. Note that these depths do not represent the swimming depths of satellite-tagged sharks (see Supplementary Figs S13 & S14), but the depth of water below the shark when the satellite location was obtained while the animal was at the surface.

Bathymetry data harvested from satellite fixes (Fig. 5) show that although tiger sharks utilize the full depth range of the insular shelf, including shallow habitats close to shore, the modal bathymetry depth, and likely centre of tiger shark activity, is between 50 and 100 m. Male average bathymetry profiles (n = 6) suggest they favour habitats further offshore than females (n = 26) (Fig. 5). For example, on average 50% of female satellite locations occurred within the 100-m depth contour, whereas on average only 28% of male satellite locations occurred across the same depth range. Note that these bathymetry depths do not represent the swimming depths of satellite-tagged sharks (see section below), but the depth of water below the shark while it was at the surface.

#### Swimming depth and temperature data from SPLASH tags

**Figure 6:**
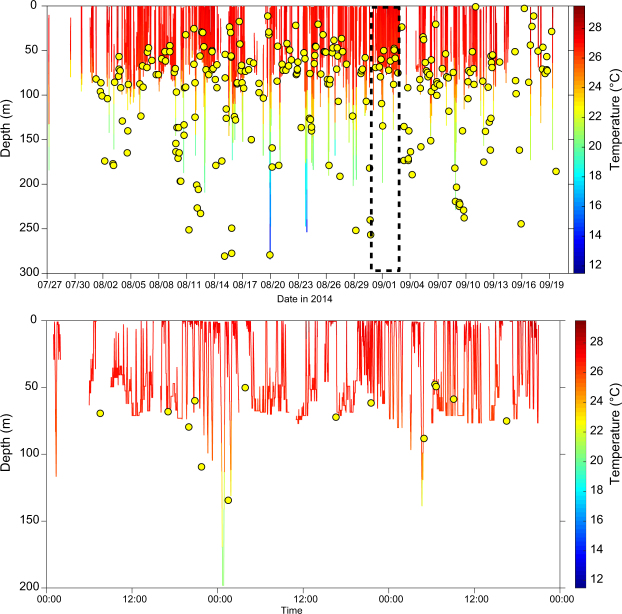
Depth and temperature time-series data from SPLASH-tag equipped tiger shark 132062. Top panel: Overview of entire time series. Bottom panel: Detail of 72 h sample of larger time series (from dashed box in upper panel). Yellow points are harvested bathymetry values (water depth below shark estimated from surface positions).

Two female sharks equipped with depth and temperature-logging dorsal fin satellite transmitters (SPLASH tags) exhibited yo-yo vertical swimming profiles, bouncing repeatedly between the surface and depths of around 100 m, with occasional deeper dives to depths of 250 m and temperatures below 15 °C (Fig. 6; Supplementary Fig. S3). Swimming depth frequency histograms indicate these two tiger sharks respectively spent 10% and 20% of their time within 2 m of the surface (Supplementary Fig. S3), and satellite location-harvested bathymetry depths from the same individuals suggest that both sharks were typically ranging between the surface and the sea floor (i.e. their bounce dives terminated at or close to the sea floor - Fig. 6).

### Acoustic monitoring results

#### 2013–2015 Maui-Oahu acoustically-tagged sharks

From 2013–2015, twenty-five receivers deployed around Maui and Oahu detected 35 (85%) of 41 acoustically-tagged tiger sharks over periods ranging from 8 to 613 days (Supplementary Table 2). Six (40%) of 15 Oahu-tagged sharks were detected on Maui receivers, but none of the 26 Maui-tagged sharks were detected on Oahu receivers. This is particularly noteworthy because tagging dates for Maui-tagged sharks were earlier on average (mean tagging date 1/19/2014) than Oahu-tagged sharks (mean tagging date 9/29/14), whereas acoustic monitoring around Oahu began slightly earlier overall (mean start date 5/20/2014) than around Maui (mean start date 5/24/2014). This resulted in a total number of potential detection days (the sum of total monitoring days for each transmitter at each receiver station) for Maui-tagged sharks on Oahu receivers (149,308 d) that was actually higher than the total number of potential detection days for Maui-tagged sharks on Maui receivers (132,761 d), and more than double the total number of potential detection days for Oahu-tagged tiger sharks around Maui (57,638 d).

Raw detection frequencies of tiger sharks around Maui and Oahu are likely skewed by the higher number of individuals tagged around Maui (26 versus 15 around Oahu), even though Maui and Oahu-tagged sharks were free to swim to other islands (and in the case of Oahu-tagged sharks, 40% did swim to Maui, and increased detection metrics for that island). Despite these limitations, raw detection frequencies provide a basic sense of the relative shark presence around each island. For example, more than half of the receiver sites around Maui detected acoustically-tagged tiger sharks every other day on average, whereas around Oahu this 50% detection day threshold was only exceeded at 1 receiver site. Individual Maui sites also had higher detection day frequencies than Oahu sites. For example, at least one tagged tiger shark was detected at Maui deep, low-recreational-use sites off Kalama and Palauea on >90% of days monitored, >80% of monitored days at Makena, 79% and 62% of monitored days at the Kalama shallow and Palauea shallow sites respectively. The shallow receiver stations at Makena, Kalama and Palauea are all high-use ocean recreation sites. Around Oahu, the highest detection day frequency was 55% and occurred at a deep water site off the north coast. Oahu shallow water, high-recreational-use sites all had detection day frequencies <6%. If we assume that these detection frequencies are approximately proportional to the number of sharks tagged around each island, doubling the frequencies observed around Oahu (equivalent to 30 tagged tiger sharks around Oahu) would still result in shallow site detection day frequencies well below those observed around Maui.

A similar pattern was evident in daily detections of multiple tiger sharks at receiver sites around Maui and Oahu. Multiple tiger sharks were detected on the same day on all Maui receivers on between 1 and 66% of days monitored, with the highest incidences of daily detections of multiple individuals at high-recreational-use shallow water locations occurring at Makena (51%), Kalama (47%), Palauea (29%) and Olowalu (25%). The highest number of tagged tiger sharks detected on a single day at any Maui site was 8 at the shallow Olowalu receiver on January 2, 2015 (Supplementary Fig. S4). Detections of multiple tagged tiger sharks only occurred on 4 Oahu receivers on between <1 and 20% of monitored days, and the highest number of tiger sharks detected on one day was 3 at a deep water site off the north coast. There were no detections of multiple tiger sharks on the same day at any Oahu shallow ocean recreation sites.

#### Legacy sharks

Five (25%) of 20 legacy tiger sharks were detected on our Maui receivers from 2013–2015 (Supplementary Fig. S5). All 5 sharks were originally captured and tagged off Oahu, with 4 individuals tagged in 2009, and one in early 2013 (Supplementary Table S3). During the current study, three of the legacy sharks were detected around both Maui and Oahu, and the remaining two were only detected around Maui (Supplementary Fig. S5). Legacy sharks were detected on 11 (79%) of 14 Maui receivers over periods ranging from 2 to 53 days (Supplementary Table S3). One male was already large enough to be sexually mature at tagging in 2009, but the remaining 4 females were sub-adults at tagging (Supplementary Table S3). A previous NOAA-sponsored project maintained an array of acoustic monitors in Maui waters from August 2008 to July 2010 (F. Parrish, unpublished data). During this earlier window of monitoring, one of the 5 legacy sharks (a sub-adult female) was detected around Maui, whereas the remaining 3 individuals (one shark was not tagged until early 2013) were only detected around Oahu (Supplementary Fig. S5). We used a Hawaii tiger shark age and growth curve^8^ to estimate the age of each of the legacy sharks at tagging, and then to estimate their age and size at the date of their first detection around Maui during the current (2013–2015) project. All 5 legacy sharks were estimated to be sexually mature when first detected around Maui during the current 2013–2015 project (Supplementary Table S3).

#### Site fidelity to receiver locations

**Figure 7:**
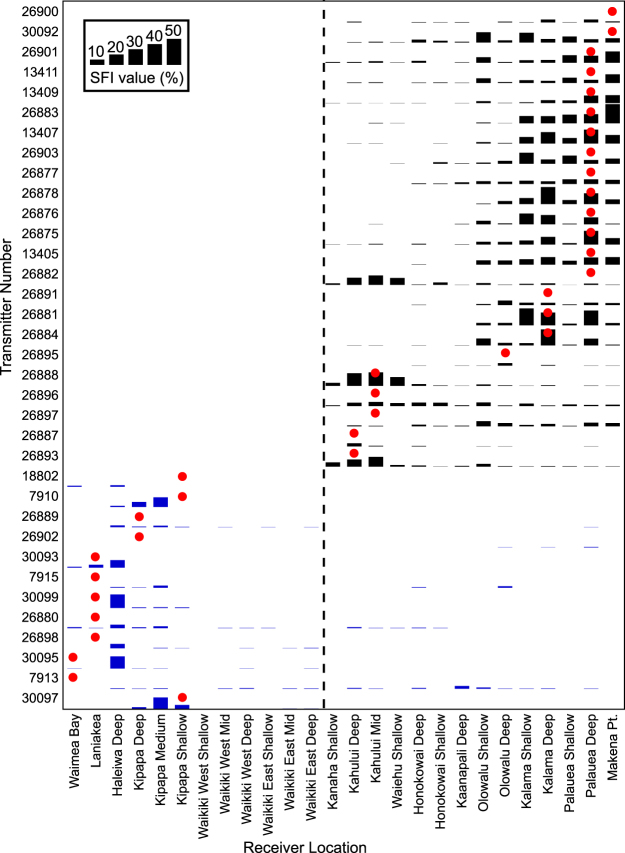
Matrix plot illustrating Site Fidelity Index (SFI) values for all Maui and Oahu-tagged sharks on all Maui & Oahu receivers. The blue bars indicate sharks tagged around Oahu, black bars are sharks tagged around Maui. Red points indicate the receiver station closest to the capture/tagging location of each shark. The dashed line bisecting the plot indicates the boundary between islands (Oahu receivers to the left, Maui receivers to the right).

Site Fidelity Index (SFI) is the percentage of monitored days on which a tagged shark was detected (see Methods for details). SFI values ranged among individuals and receiver sites from 0 to 37.9%, and the highest SFI values for each individual were often spatially-clustered among adjacent receivers (consistent with tiger shark use of home ranges with well-defined core areas – Fig. 7). Some receiver sites (e.g. Palauea Deep; Fig. 7) had relatively high SFI values for multiple sharks (suggesting core use areas of multiple individuals were overlapping at this location). Tagging-island had a significant influence on both mean and maximum SFI values (highest SFI values were typically seen around each individual’s respective tagging island), and post hoc testing revealed significant differences in site fidelity between all islands except FFS versus Hawaii, and Maui versus Oahu (Supplementary Table S6), suggesting a broad dichotomy in tiger shark site fidelity among islands, with relatively high mean SFI values observed around Maui and Oahu, and relatively low mean SFI values observed around Hawaii Island and at FFS. For example, sixteen (84%) of 19 observed SFI values exceeding 20% (equivalent to a shark visiting at least once every 5 days) were at sites around Maui, with the remaining three at sites around Oahu (Fig. 7). By island, the highest SFI values observed for any shark at any receiver site were 37.9% (Maui), 26.6% (Oahu), 17.4% (FFS) and 6.1% (Hawaii Island). The highest SFI value observed (37.9% at Makena Pt., Maui) is equivalent to this shark (a 246 cm TL female) visiting Makena every 2–3 days. Sixteen (64%) of 25 Maui-tagged sharks had SFI values >10% (equivalent to at least one visit every 10 days) at multiple receiver sites, including inshore sites at Makena, Palauea and Kalama (Fig. 7), whereas SFI values >10% were only documented in 33% of Oahu-tagged sharks, 11% of FFS-tagged sharks and none of the Hawaii Island-tagged sharks.

We used mixed models to explore the influence of spatial and biological factors (shark size and sex) on shark site fidelity. We used a hurdle approach which divides the analyses into separate presence/absence (i.e. what factors determine whether or not a shark is ever detected?) and proportional (i.e. when a shark is detected, what factors influence how often it is detected?) components. Absolute Pearson correlation coefficients were <0.29 and variance inflation factors (VIF) were <2.3 suggesting low collinearity between predictor variables. Whether or not a tiger shark was detected at a receiver site was significantly influenced by the distance between the shark tagging location and receiver site (probability of presence declined with increasing distance from tagging site) for both the best-fitting, two- and four-island GLMMs (Supplementary Tables S7 and S8; Supplementary Fig. S6). Four-island models suggested tagging island and the interaction between receiver depth and distance between the shark tagging location and receiver site significantly influenced whether or not a tiger shark was detected at a receiver site (Supplementary Table S8). Sharks tagged around Oahu and Maui had a significantly higher probability of detection than sharks tagged at FFS (Supplementary Table S8; Supplementary Fig. S6), possibly due to the overall shallower depth distribution of receivers at FFS. In addition, there was a significant interaction between receiver depth and distance between tagging location and receiver site, with a higher probability of detection at deeper sites near tagging locations (Supplementary Table S8; Supplementary Fig. S6). However, effects of receiver depth on detection probability in the four-island models need to be interpreted with caution because of the narrower, shallow-skewed depth range of receiver deployments at FFS and Hawaii Island compared to Maui and Oahu (Supplementary Table S9). The final two-island model explained 41.5% of deviance in the probability of detection by tiger sharks; however, the final four-island model explained only 23.7% of deviance indicating there are additional factors beyond this model explaining a significant proportion of their presence/absence around all four islands.

The best-fitting, two-island GAMM indicated detection frequency was significantly influenced by sex and distance between tagging location and receiver site (Supplementary Table S10). Male site fidelity was significantly lower than for female sharks, and SFI values generally decreased with increasing distance from tagging site. However, there was an increase in SFI at a distance of approximately 75–100 km caused by sharks tagged around Oahu migrating to Maui. Four-island models suggested tagging island, distance between the shark tagging location and receiver site, and receiver depth significantly influenced SFI values (Supplementary Table S11; Supplementary Fig. S7). Sharks tagged around Hawaii, Oahu, and Maui had significantly higher SFI values than FFS, and demonstrated a similar bimodal effect of distance between tagging location and receiver site as the two-island model. SFI values increased at receiver sites deeper than 30 m but shallower than 150 m. Final models explained 48.6% and 47.6% of deviance in the detection frequency of tiger sharks for two and four-island models respectively.

#### Frequency and duration of visits to receiver locations

Visits by individual tiger sharks to receiver sites were typically brief (overall average = 13.6 min, 95% of visits <30 min) and relatively frequent (over half of the intervals between successive visits were less than 48 h in duration), with local detections dominated by the most resident individuals. For example, the most frequent visitors to Maui ocean recreation sites at Kalama and Makena Pt. were detected at these locations every other day on average throughout the 2.5 year monitoring period. Maximum documented visit duration was 700.7 min (at Kalama, Maui), but visits exceeding 1 h at any site were very rare overall, accounting for only 1.3% of all documented visits.

Tagging island had a significant influence on both frequency and duration of tiger shark visits to receiver sites (Supplementary Tables S12 and S13). Both the overall mean and average maximum number of daily visits were higher for sharks tagged off Maui (overall mean = 1 visit every 12.8 days, average max = 4.6 visits per day) than any other island, but only significantly higher than for sharks tagged around Hawaii Island and FFS (Supplementary Table S12). Mean frequency of daily visits was significantly higher for sharks tagged around Oahu than sharks tagged either at FFS or off Hawaii Island, but there were no significant differences in the average maximum number of daily visits to receiver sites between sharks tagged at FFS, Oahu or Hawaii Island (Supplementary Table S12). Average duration of visits to receiver sites was in the 10–20 min range for all 4 islands, and average maximum visit duration ranged from 35 min (Hawaii Island) to 84 min (Maui; Supplementary Table S13). Post hoc testing revealed significant differences in mean visit duration between all islands except Maui and Oahu, whereas significant differences in average maximum visit duration only occurred between Maui and Hawaii, and Maui and FFS (Supplementary Table S13).

The best-fitting, two-island GAMM indicated mean visit duration was significantly influenced by sex and receiver depth (Supplementary Table S14). Males had significantly lower mean visit durations than female sharks, and mean visit duration was longest at deeper receiver sites. Four-island models suggested mean visit duration significantly increased with receiver depth (Supplementary Table S15; Supplementary Fig. S8). However, effects of receiver depth on mean visit duration in the four-island models need to be interpreted with caution due to differences in array depths among islands (Supplementary Table S9). Final models explained 34.3% and 26.4% of deviance in the mean visit duration of tiger sharks for two and four-island models respectively. Model residuals were non-normally distributed, hence results must be interpreted with caution.

### Time series results

#### Diel patterns

Tiger sharks were detected at shallow ocean recreation sites around Maui at all times of the day and night, whereas shark bites only occurred during daylight hours, with 71% of bites occurring between 0900 and 1600 (Supplementary Fig. S9). Kolmogorov-Smirnov two-sample tests indicated that the diel frequency of sharks detected was not significantly (P > 0.05) different from the null frequency (an equal number of sharks detected across all hour bins) at any of the Maui ocean recreation sites, indicating no diel influence on the overall numbers of sharks visiting these locations.

#### Seasonal patterns

Kolmogorov-Smirnov two-sample tests indicated no significant (P > 0.05) influence of season on the number of Maui-tagged tiger sharks detected around Maui, whereas there was a significant (*D*_*12,12*_ = 0.8333, P < 0.001) difference between the observed monthly frequency of Oahu-tagged tiger sharks detected around Maui and the null frequency (an even number of sharks detected each month). The number of Oahu-tagged tiger sharks detected around Maui had a distinct winter peak coinciding with tiger shark mating season (Supplementary Fig. S10). The number of Oahu-tagged sharks detected around Oahu exhibited a slight dip corresponding to the January peak seen around Maui (Supplementary Fig. S10), suggesting winter movements of Oahu sharks to Maui.

### Camera results

**Figure 8:**
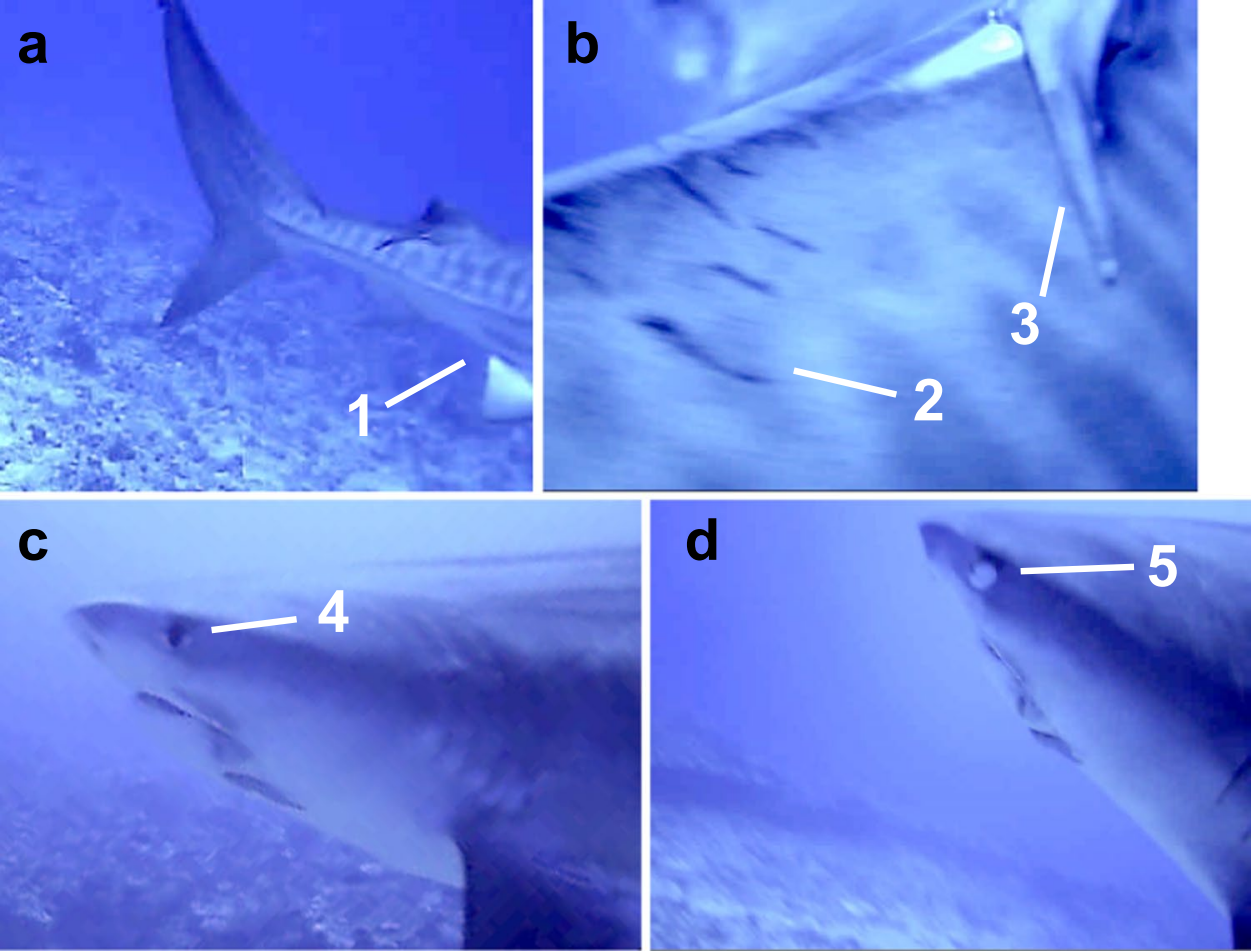
Frame grabs from video recovered from shark-mounted camera deployed on 436 cm TL, sexually mature male tiger shark captured off Oahu in January 2015. (**a**) Rear of tiger shark approached by camera shark showing no evidence of claspers on the pelvic fins (1), indicating this shark is female. (**b**) View of dorsal surface showing evidence of mating scars (2) behind the trailing edge of the dorsal fin (3). (**c**) Profile view of female tiger shark as camera shark approaches, showing nictitating membrane is retracted (4). (**d**) As camera shark makes closer approach to female tiger shark, nictitating membrane can be seen entirely-covering the eye (5). See also Supplementary video.

The shark-mounted video camera was deployed on a large (436 cm TL), sexually mature male tiger shark captured off Kaneohe Bay (Oahu) in January 2015, during mating season for tiger sharks in Hawaii. The shark had fresh abrasions on one clasper suggesting that it been actively mating shortly before capture. The camera successfully filmed for 11 h (0710–1810) on day 3 (Jan 11, 2015) of the deployment, before detaching from the shark, and surfacing off the northern tip of Oahu (Kahuku Point) at approximately 2325 (time of first satellite fix on the floating package) in an area that satellite tracking results identified as a core home range area for several Oahu-tagged tiger sharks (see example in top-left panel Fig. 3). Most of the camera footage showed the shark swimming slowly and steadily, ranging vertically between the surface and benthic habitats in which a wide variety of coral reef fishes were visible. The shark made no attempt to chase any of these fishes, nor did the fishes show any obvious flight behaviour. At 1243 the shark suddenly accelerated along the bottom in a straight-line, burst-swimming event lasting 22 s, and culminating in tight circling close to the bottom for 22 s. At the end of this spiral swimming behaviour, an apparently mature (based on size relative to other natural features in the video) female tiger shark came into camera view at close proximity to the male shark (Fig. 8). The male tiger shark swam briefly up over the dorsal surface of the female, with the camera revealing apparent mating scars behind her dorsal fin (Fig. 8). The female shark appeared to avoid contact with the male, and displayed a nictitating membrane response during the brief (8 s), close-proximity encounter (Fig. 8). The male then continued to follow the female for a further 5.85 min, during which the camera recorded brief additional glances of her in the distance. The total encounter (start of burst swimming to final image of female) lasted 6.75 min. The recorded interaction between the male and female tiger shark was consistent with an unsuccessful mating attempt.

## Discussion

Tiger shark movement patterns observed during the current study broadly match those seen during previous studies conducted in Hawaii and elsewhere throughout the species geographic range. Tiger sharks captured around Maui and Oahu exhibited a combination of wide-ranging movements (including movements between islands and extensive open-ocean excursions) and high site-fidelity to coastal habitats around ‘home’ islands. Wide-ranging movements, including open-ocean crossings of thousands of km, have been previously documented in tiger sharks captured in Hawaii^4,8^ and elsewhere in the Indo-Pacific^9–11^ and Atlantic regions^12–16^. We observed long (multi-month), open-ocean residence times for some Maui and Oahu-tagged individuals, and similar duration use of open-ocean habitats has also been previously documented in tiger sharks tagged at FFS^4^, and at Bimini (Bahamas) and Challenger Bank (Bermuda) in the Atlantic Ocean^13,15^. Tiger sharks are also captured in high-seas longline fisheries in both North Pacific^17^, and Atlantic Oceans^12,18,19^ providing further evidence of the species widespread use of open-ocean habitats.

Although tiger sharks clearly utilize open-ocean habitats in both Atlantic and Pacific oceans, the majority (78.7%) of satellite location estimates for tiger sharks captured around Maui and Oahu during the present study were in coastal waters over insular shelf habitat. Tiger shark affinity for coastal waters and shelf habitats has been documented in previous studies in Hawaii^3,4^, Australia^10^, the Galapagos^20^, the Bahamas and Florida^13^, although movements are generally much wider-ranging over the extensive continental shelf areas off Australia and Florida, than over the smaller insular shelf habitats surrounding the oceanic islands of Hawaii or the Galapagos. Conversely, most tiger sharks tagged at Challenger Bank (Bermuda, Atlantic Ocean) predominantly used open-ocean habitat^15^. These apparently conflicting patterns of open-ocean versus coastal habitat use by tiger sharks may partly stem from the demographic characteristics of sharks tagged in these different studies. For example, eighteen (95%) of 19 highly-migratory tiger sharks tagged at Challenger Bank were mature males^15^, whereas tiger sharks tagged during the current and previous Hawaii studies were mainly (59–80%) mature females which predominantly utilized coastal habitats^3–5^, and tiger sharks exhibiting high coastal residency in the Galapagos were predominantly (85%) sub-adult females and males^20^. These differences in habitat use are consistent with habitat segregation among different life history stages, with sub-adults of both sexes and mature females occupying coastal areas, and mature males occupying offshore habitats^5,8^, but likely also reflect the species ability to adapt the scale and pattern of movements to local resource distribution.

During the current study, we documented seasonal patterns of tiger shark movement, with sexually-mature individuals originally captured around Oahu visiting Maui most frequently in winter during peak mating season^6^. A previous analysis of tiger shark movements in Hawaii also found inter-island movements between MHI locations (Oahu, Maui and Hawaii Island) peaked during winter, and additionally identified migrations from the North West Hawaiian Islands (NWHI) to the MHI coincided with tiger shark pupping season during fall^5^. We found no evidence of a fall migration from the NWHI to Maui or Oahu, but this is likely due to the very low number of remaining NWHI legacy sharks (3 individuals; Supplementary Table S3) with active transmitters during the current study (i.e. fall migrations from the NWHI could have occurred undetected by our receivers). Seasonal migrations by tiger sharks have also been documented in the Atlantic^15^, Indian Ocean^21^ and Pacific^10^, and appear to be linked to sea surface temperature, especially in more temperate regions toward the latitudinal limits of tiger shark distribution^10,15^.

Although Oahu-tagged tiger sharks undertook seasonal migrations to Maui, most Maui-tagged sharks were highly site-attached to the insular shelf surrounding Maui Nui for the 21-month duration of monitoring. In fact, tiger sharks tagged around Maui typically exhibited greater residency and smaller home ranges than those tagged around other Hawaiian Islands. Site-fidelity to core islands (typically the island of capture) has been previously described for tiger sharks captured at several Hawaiian Islands^4,5^, but detailed data were not previously available for tiger sharks captured around Maui. Our current analyses suggest the home ranges of Maui-tagged tiger sharks tend to be largely contained within the extensive insular shelf habitat surrounding Maui Nui, and these home-ranges are generally smaller, with more frequently-utilized core areas, than those of tiger sharks captured around other Hawaiian Islands. Tiger shark home range core areas around Maui are closer to high-use ocean recreation sites than equivalent core areas documented around Oahu. This was clearly reflected in patterns of acoustic detections of tiger sharks at ocean recreation sites. For example, the most frequent visitors to Maui ocean recreation sites at Kalama and Makena Pt. were detected at these locations every other day on average throughout the 2.5-year monitoring period. Moreover, overall frequency of tiger shark detections (proportion of monitored days on which any electronically-tagged tiger shark was detected) was higher at monitored ocean recreation sites around Maui (62–80%) than Oahu (<6%). This disparity held true even when accounting for the fact that in this study fewer sharks were tagged around Oahu (15 sharks) than Maui (26 sharks). Assuming our tagged sharks are only a subset of all tiger sharks in Hawaii coastal waters, our detection frequencies suggest a daily, or near-daily, presence of large tiger sharks in waters adjacent to ocean recreation sites in Maui (especially SW Maui).

Overall, our results bolster existing evidence of higher tiger shark residency at isolated oceanic islands compared with locations on, or close to, extensive continental shelves. For example, high residency was also observed in tiger sharks captured at the remote Chesterfield Islands (Coral Sea)^11^ and in the Galapagos Marine Reserve^20^. By contrast, transient behaviour was observed in tiger sharks captured on wide continental shelf habitats in Australia^10,11^ and Florida^13^, or at islands close to continent shelf habitats (e.g. Bahamas^13^).

Inter-island and regional differences in tiger shark residency and home range characteristics probably reflect different patterns of resource distribution among locations. Primary resources for tiger sharks include food, conspecifics (for mating) and suitable pupping habitats, and the high residency of tiger sharks observed around Maui suggest all of these resources are available on the extensive insular shelf surrounding the islands of Maui Nui. Reef-associated organisms form the bulk of tiger shark diet in Hawaii^22^ and elsewhere^23^. In Hawaii, coral reef habitats are found to depths of at least 130 m^24^, and their horizontal distribution probably broadly mirrors that of the insular shelf^25^. As a consequence, the extensive Maui Nui insular shelf (56% of all MHI shelf habitat – Supplementary Table S1), which has the highest marine primary productivity of any Pacific island location^26^, and is known to support high densities of coral reef fishes^25^, may be able to support a larger number of tiger sharks than the smaller areas of shelf habitat surrounding the other MHI. In terrestrial systems, predator home range size is inversely-related to prey density (i.e. home ranges are smaller when prey density is high) in a variety of animals including African lions (*Panthera leo*)^27^, Eurasian lynx (*Lynx lynx*,)^28^, and northern spotted owls (*Strix occidentalis caurina*)^29^. The generally-smaller tiger shark home ranges and overlapping core areas observed around Maui may indicate higher habitat quality (i.e. prey density and availability of mates) on the extensive Maui Nui shelf than around the other MHI.

Seasonal influxes of tiger sharks from adjacent islands and as far away as the NWHI suggest the extensive Maui Nui shelf habitat is also attractive to tiger sharks from elsewhere in Hawaii, and may be an important ‘hub’ for tiger sharks within the Hawaiian chain. These migrations peak during both the fall pupping season^5^ and the winter mating season^5,6^ for tiger sharks in Hawaii, suggesting reproduction may be a factor driving these seasonal movements. If the Maui Nui shelf does in fact support more tiger sharks than other Hawaiian Islands, this could enhance the probability of encountering conspecifics during mating season, especially where home range cores overlap. Footage of a possible mating attempt from our shark-mounted camera provides further evidence that mating may be occurring in core use areas.

Although routinely detected in shallow areas, tiger sharks primarily occupy deeper waters (50–100 m depth) when they are over the insular shelf. They are vertically dynamic, making repeated “yo-yo” dives between the seabed and the surface, behavior that has been extensively documented in previous studies in Hawaii^2,29^, Australia^10^ and the Atlantic Ocean^14^. In all cases, tiger sharks are highly surface-oriented, spending 10–20% of their time within 2 m of the surface around Maui during the current study, the majority of their time at depths of < 20 m in waters off Australia^10^ and up to 51% of their time within the upper 5 m of the water column in the Atlantic Ocean^14^.

Overall, our results suggest the extensive Maui Nui insular shelf is an important natural habitat for Hawaii tiger sharks, and consequently large tiger sharks are routinely and frequently present in the waters off ocean recreation sites around Maui. This may partly explain why Maui has had more shark bites than other MHI (Supplementary Fig. S11). However, we cannot exclude unquantified differences in the numbers of ocean recreation activities between Maui and other islands as the primary cause of inter-island differences in shark bite rates. Future studies using shark-mounted video cameras and accelerometers could determine whether the Maui Nui shelf attracts tiger sharks for both reproduction and feeding. Quantifying recreational ocean use around the MHI is necessary to further clarify how these activities contribute to observed patterns of shark bites.

Despite the natural presence of large sharks in waters around Maui, the risk of shark bite remains relatively low and variable between years (Supplementary Fig. S2). Notably, 2015 saw only 2 unprovoked shark bites in Maui waters (compared to 5–8 bites in 2013–2014) even though our tracking data unequivocally show the same large, tagged tiger sharks visiting Maui ocean recreation sites regularly throughout 2013, 2014 and 2015. The relatively rarity of shark bites despite near-daily visits by large tiger sharks to high use recreation sites, suggests tiger sharks are mostly disinterested in, or even actively avoiding, people. Hawaii historically culled tiger sharks to allay public fears about shark bites but this strategy was demonstrably ineffective, with more shark bites occurring in the aftermath of culling than before^30^. A more pragmatic approach to shark bites is to allow the public to make informed choices about ocean recreation by raising awareness of the natural presence of tiger sharks in Hawaii waters (equivalent to informing people of predator presence in terrestrial wilderness habitats such as North American forests^31,32^) while stressing the low risk of shark bites, and explaining how individuals can reduce their own risk (e.g. by swimming in a group rather than alone).

## Methods

### Study area

The Hawaiian archipelago stretches 2,580 km along a SE-NW axis in the central north Pacific (Supplementary Fig. S12). The upper (NW) 1,955 km of the chain is a series of uninhabited atolls, submerged banks and seamounts, with extensive areas of photic and mesophotic (0–100 m) reef habitats. We have previously quantified tiger shark movements at these remote, uninhabited locations, and unpublished acoustic monitoring data from French Frigate Shoals atoll are included for comparative purposes in the current analyses. French Frigate Shoals (N23°45′W166°10′) is located in the middle of the Hawaiian archipelago (Supplementary Fig. S12). The atoll consists of a 34-km long oval platform bounded on the east side by a 50-km long crescent-shaped barrier reef (Supplementary Fig. S13). Habitat outside the barrier reef consists of classical spur and groove formations running from the reef crest down to depths of 20–30 m. The western half of the atoll is open to the ocean and shelves gradually from depths of 20 to 100 m over a distance of 18 km, before descending more steeply to >1000 m depth. The eastern half of the atoll consists of a shallow (<1 to 10 m deep) lagoon enclosed between the outer barrier and an inner crescent shaped reef, and is 12 km wide at its midpoint. Lagoonal habitats include reticulate and patch reefs, submerged sand and coral rubble, and small sandy islets. Total coral reef area of the shoals is >940 km^2^ and total land area of the sandy islets is 0.25 km^2^.

The lower (SE) 625 km of the Hawaiian archipelago consists of a series of 8 oceanic, high islands (Main Hawaiian Islands, MHI), of which Oahu and Maui were the focus of shark capture and tagging during this study (Supplementary Figs S12 and S13). Both Oahu and Hawaii Island have been sites of previous tiger shark research^2,3,33^ and acoustic monitoring data from these previous research efforts are incorporated in the current analyses. The MHI are surrounded by insular shelf sloping gradually from the shore out to a shelf break beginning at depths of between 100 and 200 m. The width of insular shelf varies among islands, with the Maui Nui complex (the islands of Maui, Kahoolawe, Lanai and Molokai) having a more extensive insular shelf than the islands of Niihau, Kauai, Oahu and Hawaii (Supplementary Table S11; Supplementary Fig. S13). The insular shelf contains a variety of photic and mesophotic coral reefs, macroalgal beds and sandy habitats^34^.

Maui County (the islands of Maui, Lanai, Molokai and Kahoolawe) is the second most populous in the State of Hawaii, with a similar population to Hawaii County, and double the population of Kauai County (Supplementary Table S1). All of the major MHI have well-developed public beach park infrastructure and public shoreline access, allowing easy access to the ocean for recreational activities including swimming, snorkelling, spearfishing, surfing, paddleboarding and kite surfing. These activities occur year-round and are participated in by both residents and visitors. The coastlines of each MHI include both highly-developed, heavily-used areas, and rugged, inaccessible areas where ocean recreation is much less common. The gross spatial distribution of shark bites around each island largely reflects overall spatial patterns of human recreational ocean activities (i.e. most shark bites occur at locations frequently used for ocean recreation). For example, the eastern coastline of Maui is remote, rugged and wind-exposed. Few ocean recreation activities occur along this stretch of coastline, and consequently shark bite incidents are extremely rare in this area (see Supplementary Fig. S14). Similarly the north-eastern coast of Oahu has relatively-low recreational ocean use and a low number of shark bite incidents (Supplementary Fig. S15). However, some of the most heavily-used beaches (e.g. Waikiki, Oahu) also have a low rate of shark bite incidents suggesting the number of people present in the water is not the only determinant of where shark bites occur.

### Shark capture and tagging

At all four islands (Hawaii, Maui, Oahu and FFS), we captured and restrained tiger sharks using our standard operating protocols^4^. While inverted in tonic immobility, acoustic transmitters were implanted into the peritoneal cavity of each shark through a small incision in the abdomen that was then closed using interrupted sutures. Satellite transmitters were attached to the dorsal fin of each shark^4^. Prior to release, sharks were externally tagged with unique identification tags (Hallprint, Hindmarsh Valley, Australia).

To provide a ‘sharks-eye’ view of habitat use, two individuals were also equipped with a small video camera package attached to the left pectoral fin. The camera (DVL400L, Motion JPEG, VGA [640 × 480, max. 30 fps], Little Leonardo Inc., Tokyo, Japan), embedded within a small syntactic foam float, was held in place via a fusible steel band, passed around the package and through two small holes drilled through the pectoral fin. The entire package had a forward view when deployed on the shark, and was released after 72 h by an electronic timer. The camera was programmed to begin filming around sunrise on day 3 of the deployment, and continue to film all day before detaching from the shark. Once at the surface, the camera package was located and recovered using satellite and VHF transmitters attached to the float.

Shark handling and tagging activities were carried out in accordance with the animal use protocols of the University of Hawaii Institutional Animal Care and Use Committee (IACUC) and were approved under IACUC protocol #05-053.

### Electronic tags overview

We used two types of electronic telemetry tag to quantify different aspects of tiger shark spatial dynamics; (1) Dorsal fin-mounted satellite transmitters to provide a broad overview of shark horizontal movements and habitat use patterns, and (2) Surgically-implanted, coded acoustic transmitters to provide long-term presence-absence data at specific locations monitored by underwater acoustic receivers. Some of the satellite transmitters were equipped with depth and/or temperature sensors to provide additional insight into shark vertical behaviour and thermal environment.

### Dorsal fin-mounted satellite transmitters

Three different types of dorsal-fin mounted satellite transmitters were used to quantify tiger shark horizontal and vertical movements; (1) SPOT tags (SPOT-258A, 106 mm × 45 mm × 19 mm, 53 g, Wildlife Computers, Redmond, WA, USA), which only yield Argos quality location estimates for tagged sharks, (2) SPLASH tags (SPLASH10-312A, 133 mm × 44 mm × 19 mm, 85 g, Wildlife Computers, Redmond, WA, USA) which provide an Argos-quality location together with a packet of sensor data from onboard depth, temperature and other sensors, and (3) Fastloc-GPS tags (SPOT-F-338A, 109 mm × 53 mm × 21 mm, 81 g, Wildlife Computers, Redmond, WA, USA) which capture Global Positioning System (GPS) quality positions that are then transmitted to the Argos satellite array. Fin-mounted tags transmit a signal to the Argos satellite array whenever the dorsal fin breaks the surface of the water. These transmissions yield geolocation estimates with location accuracy classes ranging from 3 to 1 (best to worst). The following root mean squares errors are provided by the Argos tracking and environmental monitoring system (www.argos-system.org), Class 3 <= 150 m, Class 2 = 150–300 m, and Class 1 = 350–1000 m. Location qualities of 0, A, B, and Z (in order of decreasing quality) are also obtained, but no estimates of error size are given for these classes. However, accurate fixes are possible with all location qualities except Z, and previous studies have shown that, with appropriate filtering, Argos location classes (LC) 0, A and B can provide useful information for tracking marine mammals^35^. Fastloc-GPS tags provide both Argos quality and GPS quality (from <5 to >724 m) positions^36^.

Argos satellite coverage averages only 6–12 minutes per hour in Hawaii, with only a subset of this coverage composed of high-quality satellite passes. To increase data recovery from SPLASH and Fastloc-GPS tags, two prototype land-based satellite receivers (Mote-system^TM^, Wildlife Computers, Redmond, WA, USA) were deployed at high elevations on Maui. These land-based receivers increased data recovery from individual satellite tags by up to 700%.

Prior to deployment, satellite tags were coated with two types of antifouling compound to prolong their functional lives. Non-conducting surfaces were coated with Propspeed^TM^ (Oceanmax Manufacturing Ltd., Auckland, New Zealand), and the wet-dry electrodes were coated with electrically conductive C-Spray antifouling compound (YSI Inc., Yellow Springs, OH, USA).

### Analysis of satellite tag data

#### Satellite data reduction and filtering

Argos locations from satellite tag-equipped sharks were filtered prior to analysis. We first manually removed obviously spurious distant locations, and then used a land-avoiding swim-speed filter to eliminate remaining low-probability class A, B and 0 locations. Our chosen swim speed threshold (4.2 km/h) was based on empirical tiger shark swimming speed data derived from previous active tracking^2^ and shark-mounted accelerometers containing speed sensors^29^, together with GPS quality locations and multi-day, highly-directional swimming events from the present study. Higher-quality locations (i.e. LC 1, 2 and 3) were used to anchor the swim speed filter. Thus LC A, B and 0 locations that lay within a 4.2 km/h buffer of a previous higher-quality location were retained, whereas those beyond the buffer were eliminated from the data set.

#### Coastal Home Range Analyses of Satellite Tracking Data

We used the T-LoCoH (Time Local Convex Hull) package^37^ in R^38^ to construct home range utilization distributions from tiger shark Argos and GPS locations. To avoid autocorrelation due to oversampling bias, we used the T-LoCoH analysis package data cleaning tools to identify temporally-clustered locations in our speed-filtered data. We then selecting a re-sampling threshold (based on median sampling interval) that reduced each cluster to a single location. Data were then transformed from Latitude-Longitude to Universal Transverse Mercator (UTM Zone 4) coordinates.

We then evaluated 3 alternative methods (k-based, r-based and adaptive) for creating hulls and density isopleths (i.e., utilization distributions). The k-based method constructs kernels from k-1 nearest neighbours of root points, the r-based method constructs kernels from all points within a fixed radius ‘r’ of each reference point, and the adaptive method constructs kernels from all points within a radius ‘a’ such that the distances of all points within the radius to the reference point sum to a value less than or equal to ‘a’.

We found that the k-based method was prone to Type I errors (including unused area outside the home range) and the r-based method prone to Type II errors (omitting area the animal used). The adaptive method provided the most robust estimates of home range utilization distribution^37^, and we selected this method for constructing tiger shark home range utilization distributions. We removed offshore migrations (i.e. those points forming obvious offshore loops extending beyond the 4000 m coastal isobath) from the analyses of coastal habitat use, as including these resulted in extensive Type I errors within coastal areas. All other locations were included in the analyses, with no time-based weighting (i.e. S = 0). The location and size of core (25%) isopleths for each shark were stable across a wide range of ‘a’ values, whereas the outer (95%) isopleths expanded with increasing ‘a’ values until they overlapped terrestrial habitat. We selected ‘a’ values that eliminated gaps within the 95% isopleth surfaces while avoiding overlap with terrestrial habitat.

#### Bathymetry profiles from Argos locations

We used the ‘marmap’ package^39^ in R to extract underlying bathymetry values for each of the speed-filtered Argos locations. These values do not reflect swimming depth (sharks must be at the surface for Argos location estimates to occur), but rather indicate the depth of the ocean floor over which the shark was detected. Habitat depth estimates were obtained from a 3 arc-second (approx. 93 m) resolution bathymetry matrix around the MHI and a 30 arc-second (approx. 926 m) resolution matrix for offshore locations.

#### Swimming depth data from SPLASH tags

To obtain insights into tiger shark vertical movements, we equipped several individuals with dorsal fin-mounted SPLASH tags that collect information on swimming depth and ambient temperature at 10 s intervals. These data are summarized (75 s and 10 min intervals) on-board the tag, and transmitted in a sequence of data packets when the dorsal fin emerges above the surface. Transmitted data were recovered via both the Argos satellite array and land-based Mote system of receivers. SPLASH tags also yield Argos locations and we used harvested bathymetry profiles (see section above) to provide a bottom depth reference for tiger shark vertical movements.

### Acoustic monitoring system

We used the VR2W acoustic monitoring system (Vemco, Bedford, Nova Scotia, Canada) to quantify tiger shark presence at specific locations around the coastlines of Maui and Oahu. This system consists of small (340 mm long × 60 mm diameter, weight in water 300 g), self-contained, single channel (69 kHz) underwater receivers which listen continually for the presence of coded-pulse acoustic transmitters. Sharks captured around Maui and Oahu between October 2013 and February 2015 were equipped with acoustic transmitters (V16-6H, 16 × 94 mm, weight in water 14 g, Vemco, Bedford, Nova Scotia, Canada) which periodically emit a ‘pulse train’ of closely spaced 69 kHz ‘pings’, uniquely identifying each shark. These pulse trains average 3 to 5 s in duration, and transmitters were silent for a randomized period of 20 to 230 s between each pulse train (Supplementary Table S2). Each successfully decoded pulse train is recorded as a single detection by a VR2W receiver, and is stored in the receiver memory as the unique transmitter number, date and time of detection.

The nominal transmitter battery lives ranged from 2 to 10 years, depending on transmission duty cycle (i.e. how often the identification code is sent). Most transmitters deployed in tiger sharks captured around Maui during the present study had 10-year nominal battery lives, and are expected to continue to transmit until 2023 and 2024 (Supplementary Table S2). Transmitters deployed in tiger sharks captured around Oahu during the Maui study, had nominal battery lives ranging from 2–10 years, with all expected to continue transmitting beyond the 2-year time frame of the Maui study (Supplementary Table S2).

In addition to tiger sharks captured and tagged for the present Maui-Oahu focused study, the Maui and Oahu acoustic arrays also listened for transmissions from 20 ‘legacy’ tiger sharks that were captured and acoustically-tagged around Oahu (n = 17), and FFS (n = 1), Lisianski Island (n = 1) and Pearl and Hermes Atoll (n = 1) in the NWHI, during earlier research projects (2009–2013). These transmitters had nominal battery lives ranging from 2–10 years resulting in both full and partial overlap of transmitter lives with the 2-year monitoring period around Maui (Supplementary Table S3).

Acoustic detection ranges were empirically determined by deploying transmitters on a weighted line from a skiff equipped with an on-board GPS-equipped Vemco VR100 receiver and hydrophone. The transmitter was first deployed directly over each receiver, and allowed to transmit 10 times before recovery. This same process was repeated at 100-m intervals beyond the receiver to a maximum distance of 1.5 km. The VR100 receiver recorded the time and position of each transmission. The originating positions of transmissions logged by the underwater VR2W receivers during range tests were subsequently determined by cross-referencing VR2W and VR100 logs. Detection range was up to 900 m.

We deployed 26 receivers around the coasts of Maui (15 receivers, of which 14 were recovered) and Oahu (12 receivers) (Supplementary Figs S14 and S15). Our array spanned the depth range of the insular shelf, with shallow (5–20 m depth), inshore units deployed at high recreational use (i.e. swimming, snorkelling and surfing) sites, including locations of recent shark bite incidents, and offshore units deployed in deeper (100–200 m depth) waters up to several km offshore (Supplementary Figs S14 and S15). This array design allowed us to compare tiger shark presence between deep and shallow areas, between different coasts of the same island, and between Maui and Oahu. The geographic spread of receivers also allowed for cross-validation of acoustic and satellite data obtained from individual tiger sharks equipped with both kinds of transmitter.

Receivers were deployed on subsurface moorings consisting of an end weight, or sand screw, 2–3 m of polypropylene rope and a hard float. Receivers were attached to the mooring rope using a combination of heavy duty nylon cable ties and stainless steel hose clamps. Shallow receivers were recovered by either SCUBA divers or snorkelers. Deep receivers incorporated an acoustic release (AR-60-E, Sub Sea Sonics LLC, El Cajon, CA, USA) between the end weight and the polypropylene rope. To recover deep receivers, a surface control box, equipped with a hydrophone, activated burn wires attaching the acoustic release to the end weight, freeing the mooring from the seabed and allowing it to float to the surface.

### Historical acoustic data sets for comparison with current results

To provide a frame of reference for current acoustic monitoring results from receiver stations around Maui and Oahu, we include two previous data sets in our analyses. These data were collected during tiger shark acoustic tagging studies conducted around Hawaii Island (2003–2005; Supplementary Table S4) and FFS (2009–2011; Supplementary Table S5). Both of these previous studies utilized the Vemco acoustic monitoring system and equipped tiger sharks with surgically-implanted V16–6H transmitters. The duty cycles of transmitters used at FFS and around Hawaii Island were faster (i.e. a higher number of transmissions per unit time) than those used in the current study, and consequently the transmitter lives were shorter (~2 years; Supplementary Tables S4 and S5).

The use of different transmitter duty cycles at different islands is a potential source of detection bias. In this case, the bias is for less frequent detection of sharks tagged around Maui compared to the other three islands, due to a combination of slower Maui transmitter duty cycles, and very low risk of signal collision reducing detection rates of faster duty cycle tags deployed around other islands, because tiger sharks rarely overlap in time and space. We mitigate (but cannot completely eliminate) any potential bias by using comparative metrics with relatively large time footprints (i.e. the longer you listen, the higher the probability you have of detecting a tag, regardless of its duty cycle). For example, we are using calendar days as the basis of our Site Fidelity Index (SFI).

The Hawaii Island monitoring array consisted of 33 receivers deployed at depths between 5–30 m on fringing reef along 115 km of the western coastline (Supplementary Fig. S16). The FFS array was comprised of 24 receivers deployed primarily across the extensive atoll lagoon, but also at several sites outside the barrier reef (Supplementary Fig. S16). Both Hawaii Island and FFS receiver arrays had narrower, shallow-skewed depth ranges compared to those deployed around Oahu and Maui (Supplementary Table S9).

### Analysis of acoustic data

#### Site fidelity and duration of visits to receiver locations

To determine whether tiger sharks captured at sites of concern around Maui show any evidence of being more resident, visiting coastal recreation sites more often, or spending more time in these areas than tiger sharks captured around other Hawaiian Islands, we compared site fidelity and visit characteristics (duration and frequency of visits to receiver locations) of tiger sharks captured around Maui, with those captured around Oahu, Hawaii Island and FFS.

SFI was calculated by dividing the number of days a shark was detected at each receiver, by the monitoring period for that site, and then expressed as a percentage by multiplying by 100^33^. The SFI assumes zero tag battery failure, and the monitoring period for each receiver-transmitter combination was adjusted to account for receivers that were deployed after sharks were tagged (i.e. monitoring period commenced with receiver deployment) and vice versa, and also to account for the anticipated battery lives of each transmitter.

The duration of tiger shark visits to each receiver location was quantified by calculating the time elapsed between the first and last transmitter detections during each visit. A visit started and ended when either the location changed, or the transmitter was not detected for 30 minutes^3^. Visits consisting of single transmitter detections were considered to last 7.7 min (equivalent to the longest transmitter pulse train duration of 3.6 s, preceded and followed by listening periods equivalent to the maximum random off time of 230 s). These buffer criteria were based on the slowest transmitter duty cycles and were applied to all transmitters included in our analyses to avoid bias resulting from faster transmitter duty cycles yielding inherently shorter visits. The time elapsed between consecutive visits was used to determine how long each shark was absent from its most frequently visited location.

For each shark, we calculated the mean (excluding zeros) and maximum SFI values, the mean and maximum visit duration (min), and the mean (excluding zeros) and maximum number of visits per day, and then used one-way analyses of variance (ANOVAs) to compare tiger shark overall average site fidelity and visit characteristics among tagging islands (preliminary analysis indicated shark sex and the interaction between sex and tagging island had no significant [P > 0.05] influence on mean site fidelity or visit characteristics). Averaging the response variables by individual shark was necessary to meet the assumption that observations are independent of one another. Box-Cox transformations were used to normalize each dataset prior to testing, but failed to produce normally-distributed residuals for maximum number of visits per day, therefore a nonparametric Kruskal-Wallis test with Wilcoxon rank-sum test was used to compare means of this variable among tagging islands. Following Box-Cox transformation, data were tested for equality of variance using Levene’s test and Bartlett’s test. All variables except maximum SFI were heteroscedastic, thus a conventional one-way ANOVA (with Tukey’s HSD test) was used to compare average maximum SFI values among islands, and Welch’s ANOVA (with Games-Howell test), which accounts for unequal variance when comparing means, was used to compare the tagging-island averages of the remaining site fidelity and visit characteristic variables.

We used mixed models to explore how site fidelity and visit characteristics were influenced by shark sex, size and tagging island, and receiver depth and distance from tagging site. Prior to fitting models, data exploration was carried out per Zuur *et al*.^40^. Collinearity between candidate predictor variables was assessed using Pearson correlation coefficients and VIFs using the ‘corvif’ function in R (http://www.highstat.com/book2.htm)^40,41^. Transmitter (i.e. the individual shark) and receiver station were included as random effects to account for the repeated measures associated with multiple detections of the same individuals on each receiver.

Exploratory data analyses revealed the raw SFI detection data were extremely (>70%) zero-inflated and highly-skewed. The zero-inflation was a by-product of a receiver array that extended beyond the home ranges of individual sharks. To account for this, we used a hurdle model approach^41,42^, where the binary (presence/absence) and proportional (i.e. non-zero) components of the data are analysed separately. This technique essentially poses two questions; (1) What factors determine whether or not a shark is ever detected, and (2) When a shark is detected, what factors influence how often it is detected?

For the binary component of the hurdle model, we used generalized linear mixed-effects models (GLMMs) with a binomial distribution and logit link function constructed in R using the ‘lme4’ package^43^. Site fidelity data were converted to zeros (never detected on a receiver) and ones (detected on a receiver). The total number of days that each receiver was actively listening for each tagged shark was included as an offset term in the models. Candidate predictor variables included the distance between shark tagging site and receiver location, receiver station depth, shark sex, total length and tagging island, and the interactions between sex and depth and between tagging site-receiver distance and depth. The justification for selecting these interaction terms were *a priori* evidence of sex segregation among mature tiger sharks (males further offshore than females), and core-structured home ranges (receivers at preferred depths but at the periphery of the home range will have low SFI values). Interactions were only considered in models containing both main effects (i.e. rule of marginality). Continuous predictor variables were mean centred and scaled prior to analyses^44^.

Exploratory data analyses revealed non-linear relationships between site fidelity (excluding zeros) and visit characteristics, and candidate explanatory variables. Therefore, these data were analysed using generalized additive mixed models (GAMMs) constructed in R using the ‘gamm4’ package^45^. The proportional (i.e. non-zero) component of site fidelity was modelled using a binomial distribution with a logit link function. Proportional SFI values were converted to the ratio of the total number of days detected at each receiver station to the total number of days in which there were no detections according to the unique number of days that each receiver was actively listening for each tagged shark.

We used mean visit duration (rounded to the nearest integer) as our response variable in analyses of factors influencing visit characteristics at receiver sites. We used a Kolmogorov-Smirnov two-sample test to compare the original and rounded values to ensure that integer smoothing did not significantly (P > 0.05) alter the distributional properties of the visit duration data. Mean visit duration was modelled using a gamma distribution with a log link function. GAMM analyses included the distance between shark tagging site and receiver location, receiver depth, shark sex, total length and tagging island as candidate predictor variables. Models were explored using unrestricted smooths, but final models limited the basis (*k*) used to represent the smooth terms at 5^46^.

Model selection was based upon an information-theoretic approach through minimization of the Akaike Information Criterion corrected for small sample size (AIC_*c*_)^47^ using the ‘MuMIn’ package^48^. Models with substantial support were selected based on a ΔAIC_*c*_ < 2 from the model with the lowest AIC_*c*_ and included in model averaging based on Akaike weights (*w*)^47^. A single best model containing only predictor variables with high relative importance was used for graphical representation, calculation of deviance explained, and evaluation of model fitness and adherence to statistical assumptions of residuals.

The general framework described above was initially applied to two-island models (Maui and Oahu data only), and then subsequently to four island models (Maui, Oahu, FFS and Hawaii Island). We subdivided analyses in this way because tagging and monitoring on Maui and Oahu were contemporaneous (i.e. all sharks tagged around Maui and Oahu were in theory detectable by all receivers deployed around both islands), whereas FFS and Hawaii Island data were historical and non-overlapping (i.e. sharks tagged at these islands could only be detected on the arrays at the tagging islands due to the timing of the studies and transmitter battery life constraints), and these islands also had receivers arrays deployed across a narrower depth range (1–46 m) than Maui and Oahu (8–195 m; Supplementary Table S9).

#### Temporal Patterns

We used graphical techniques to identify temporal (diel and seasonal) patterns in the total number of tiger sharks visiting receiver sites. Detection data were pooled across the 21-month monitoring period and aggregated into hourly and monthly bins to determine how the number of individuals detected varied at both diel and seasonal scales. Seasonal patterns were evaluated at island level (Maui versus Oahu) and diel patterns were examined at each of the shallow ocean recreation sites around Maui. We assumed a null pattern (i.e. no diel or seasonal effect on frequency of detection) would be represented by an equal (the mean) number of individuals detected in all diel or seasonal bins, and used Kolmogorov-Smirnov two-sample tests to compare the null frequency against the observed frequency of detection.

## Electronic supplementary material

Supplementary Information

Supplementary Information

## References

[1] Randall JE (1992). Review of the biology of the tiger shark (*Galeocerdo cuvier*) Australian. J. Mar. Fresh. Res..

[2] Holland KN, Wetherbee BM, Lowe CG, Meyer CG (1999). Movements of tiger sharks (*Galeocerdo cuvier*) in coastal Hawaiian waters. Mar. Bio..

[3] Meyer CG, Clark TB, Papastamatiou YP, Whitney NM, Holland KN (2009). Long term movement patterns of tiger sharks *Galeocerdo cuvier* in Hawaii. Mar. Ecol. Prog. Ser..

[4] Meyer CG, Papastamatiou YP, Holland KN (2010). A multiple instrument approach to quantifying the movement patterns and habitat use of tiger (*Galeocerdo cuvier*) and Galapagos sharks (*Carcharhinus galapagensis*) at French Frigate Shoals, Hawaii. Mar. Bio..

[5] Papastamatiou YP (2013). Telemetry and random walk models reveal complex patterns of partial migration in a large marine predator. Ecology.

[6] Whitney NM, Crow GL (2007). Reproductive biology of the tiger shark (*Galeocerdo cuvier*) in Hawaii. Mar. Bio..

[7] Jeanniard-du-Dot T, Holland K, Schorr GS, Vo D (2017). Motes enhance data recovery from satellite-relayed biologgers and can facilitate collaborative research into marine habitat utilization. Animal Biotelemetry.

[8] Meyer CG (2014). Growth and maximum size of tiger sharks (*Galeocerdo cuvier*) in Hawaii. PLoS ONE.

[9] Heithaus MR, Wirsing AJ, Dill LM, Heithaus LI (2007). Long-term movements of tiger sharks satellite-tagged in Shark Bay, Western Australia. Mar. Bio..

[10] Holmes BJ (2014). Tiger shark (*Galeocerdo cuvier*) movement patterns and habitat use determined by satellite tagging in eastern Australian waters. Mar. Bio..

[11] Werry JM (2014). Reef-fidelity and migration of tiger sharks, *Galeocerdo cuvier*, across the Coral Sea. PLoS ONE.

[12] Kohler NE, Casey JG, Turner PA (1998). NMFS Cooperative Shark Tagging Program, 1962-93: An atlas of shark tag and recapture data. Mar. Fish. Rev..

[13] Hammerschlag N, Gallagher AJ, Wester J, Luo J, Ault JS (2012). Don’t bite the hand that feeds: assessing ecological impacts of provisioning ecotourism on an apex marine predator. Funct. Ecol..

[14] Vaudo JJ (2014). Intraspecific variation in vertical habitat use by tiger sharks (Galeocerdo cuvier) in the western NorthAtlantic. Ecol. Evol.

[15] Lea JSE (2015). Repeated, long-distance migrations by a philopatric predator targeting highly contrasting ecosystems. Sci. Rep-UK.

[16] Afonso AS, Garla R, Hazin FHV (2017). Tiger sharks can connect equatorial habitats and fisheries across the Atlantic Ocean basin. PLoS ONE.

[17] Polovina JJ, Lau BB (1993). Temporal and spatial distribution of catches of tiger sharks, *Galeocerdo cuvier*, in the pelagic longline fishery around the Hawaiian Islands. Mar. Fish. Rev..

[18] Beerkircher LR, Cortes E, Shivji M (2002). Characteristics of shark bycatch observed on pelagic longlines off the southeastern United States, 1992-2000. Mar. Fish. Rev..

[19] Domingo A (2016). Is the tiger shark *Galeocerdo cuvier* a coastal species? Expanding its distribution range in the Atlantic Ocean using at-sea observer data. J. Fish Biol..

[20] Acuña-Marrero D (2017). Residency and movement patterns of an apex predatory shark (*Galeocerdo cuvier*) at the Galapagos Marine Reserve. PLoS ONE.

[21] Wirsing AJ, Heithaus MR, Dill LM (2006). Tiger shark (*Galeocerdo cuvier*) abundance and growth in a subtropical embayment: evidence from 7 years of standardized fishing effort. Mar. Biol..

[22] Lowe CG, Wetherbee BM, Crow GL (1996). Ontogenetic dietary shifts and feeding behaviour of the tiger shark, *Galeocerdo cuvier*, in Hawaiian waters. Env. Biol. Fish..

[23] Simpfendorfer CA, Goodreid AB, McAuley RB (2001). Size, Sex and Geographic Variation in the Diet of the Tiger Shark, *Galeocerdo Cuvier*, From Western Australian Waters. Env. Biol. Fish..

[24] Kahng SE, Copus JM, Wagner D (2014). Recent advances in the ecology of mesophotic coral ecosystems (MCE). Curr. Opin. Env. Sus..

[25] Rooney J (2010). Mesophotic coral ecosystems in the Hawaiian Archipelago. Coral Reefs.

[26] Loveridge AJ (2009). Changes in home range size of African lions in relation to pride size and prey biomass in a semi-arid savanna. Ecography.

[27] Herfindal I, Linnell JDC, Odden J, Birkeland Nilsen E, Andersen R (2005). Prey density, environmental productivity and home-range size in the Eurasian lynx (*Lynx lynx*). J. Zool..

[28] Zabel CJ, McKelvey K, Ward JP (2005). Influence of primary prey on home-range size and habitat-use patterns of northern spotted owls (*Strix occidentalis caurina*). Can. J. Zool..

[29] Nakamura I, Watanabe YY, Papastamatiou YP, Sato K, Meyer CG (2011). Yo-yo vertical movements suggest a foraging strategy for tiger sharks *Galeocerdo cuvier*. Mar. Ecol. Prog. Ser..

[30] Wetherbee BM, Lowe CG, Crow GL (1994). A review of shark control in Hawaii with recommendations for future research. Pac. Sci..

[31] Lackey B, Ham S (2004). Assessment of Communication Focused on Human-Black Bear Conflict at Yosemite National Park. J. Interpret. Res..

[32] Dunn WC, Elwell JH, Tunberg G (2008). Safety education in bear country: Are people getting the message. Ursus.

[33] Papastamatiou YP, Itano DG, Dale JJ, Meyer CG, Holland KN (2010). Site fidelity and movements of sharks associated with ocean-farming cages in Hawaii. Mar. Fresh. Res..

[34] Pyle RL (2016). A comprehensive investigation of mesophotic coral ecosystems in the Hawaiian Archipelago. PeerJ.

[35] Vincent C, Mcconnell BJ, Ridoux V, Fedak MA (2002). Assessment of argos location accuracy from satellite tags deployed on captive gray seals. Mar. Mammal Sci..

[36] Dujon AM, Lindstrom RT, Hays GC (2014). The accuracy of Fastloc-GPS locations and implications for animal tracking. Methods Ecol. Evol..

[37] Lyons AJ, Turner WC, Getz WM (2013). Home range plus: a space-time characterization of movement over real landscapes. Movement Ecology.

[38] R Core Team. R: A language and environment for statistical computing. R Foundation for Statistical Computing, Vienna, Austria. ISBN 3-900051-07-0, http://www.R-project.org/ (2014).

[39] Pante E, Simon-Bouhet B (2013). Marmap: A package for importing, plotting and analyzing bathymetric and topographic data in R. PLoS ONE.

[40] Zuur AF, Ieno EN, Elphick CS (2010). A protocol for data exploration to avoid common statistical problems. Methods Ecol. Evol..

[41] Zuur, A.F., Ieno, E.N., Walker, N.J., Saveliev, A.A. & Smith, G.M. Mixed Effects Models and Extensions in Ecology with R. Springer (2009).

[42] Min Y, Agresti A (2005). Random effect models for repeated measures of zero-inflated count data. Stat. Model..

[43] Bates, D., Maechler, M., Bolker, B.M. & Walker, S. lme4: Linear mixed-effects models using Eigen and S4. 2014. R package version 1.1-6, http://CRAN.R-project.org/package=lme4 (2014).

[44] Schielzeth H (2010). Simple means to improve the interpretability of regression coefficients. Methods Ecol. Evol..

[45] Wood, S. & Scheipl, F. gamm4: Generalized additive mixed models using mgcv and lme4 (v. 0.2-3), http://CRAN.R-project.org/package=gamm4 (2014).

[46] Keele, L. J. *Semiparametric Regression for the Social Sciences*. (John Wiley & Sons, Ltd, 2008).

[47] Burnham KP, Anderson DR, Huyvaert KP (2011). AIC model selection and multimodel inference in behavioral ecology: some background, observations, and comparisons. Behav. Ecol. Sociobiol..

[48] Barton, K. MuMIn: Multi-model inference. R package version 1.10.0, http://CRAN.R-project.org/package=MuMIn (2014).

