# Supplementary material for: Habitat geography around Hawaii’s oceanic islands influences tiger shark (Galeocerdo cuvier) spatial behaviour and shark bite risk at ocean recreation sites
Source: Sci Rep. 2018 Mar 21;8:4945. doi: 10.1038/s41598-018-23006-0 (PMC5862960; doi:10.1038/s41598-018-23006-0)
Supplement: Supplementary file 1 — Supplementary Information [file 41598_2018_23006_MOESM1_ESM.docx]

**Supplementary information for:**

**Habitat geography around Hawaii’s oceanic islands influences tiger shark (*Galeocerdo cuvier*) spatial behaviour and shark bite risk at ocean recreation sites**

C.G. Meyer, J.M. Anderson, D.M. Coffey, M.R. Hutchinson, M.A. Royer and K.N. Holland

**
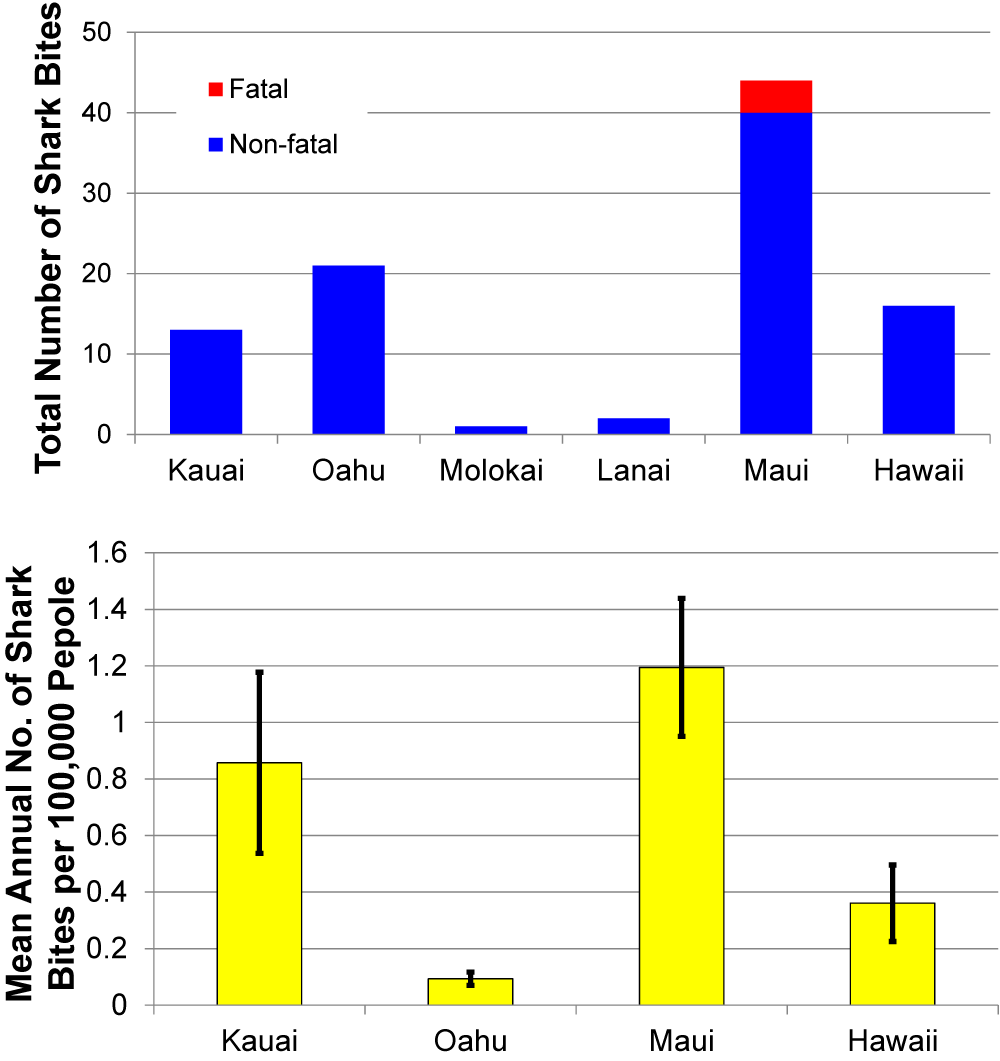
**

Supplementary Figure S1. Hawaii shark bite statistics. Top: Total numbers of unprovoked shark bites recorded on each Main Hawaiian Island 1995-2015. Bottom: Twenty year (1995-2014) average *per capita* shark bite rate (no. of unprovoked shark bites per 100,000 people) in Kauai, Honolulu, Maui (Maui county includes Molokai and Lanai) and Hawaii counties. Error bars are ± standard error. Note: the *per capita* estimates are based on *de facto* population size estimates which combine Hawaii residents and visitors. Sources, DLNR-DAR and Hawaii Department of Business, Economic Development and Tourism (DBEDT).


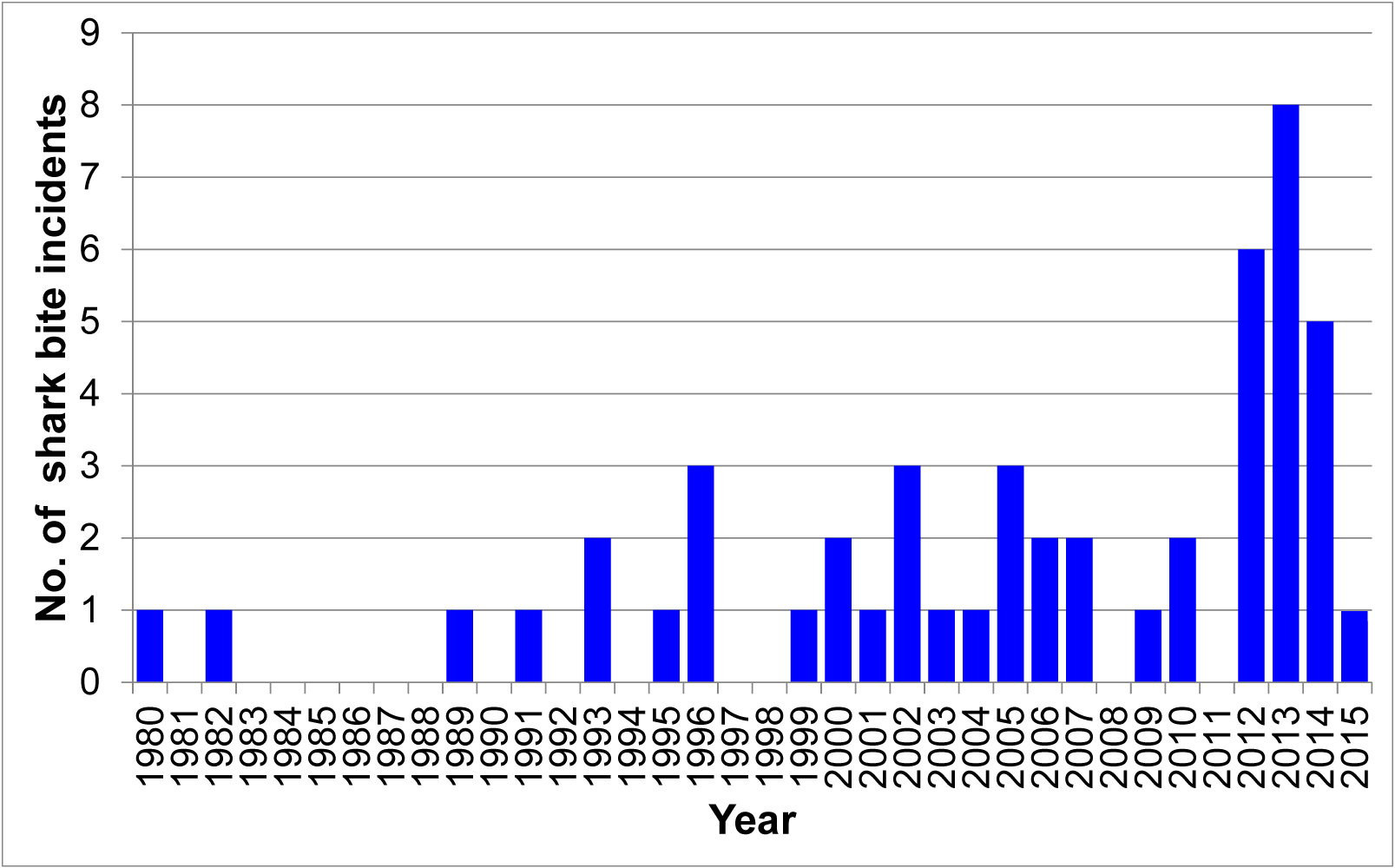


Supplementary Figure S2. Annual numbers of shark bite incidents around Maui island, 1980-2015. Source Hawaii Department of Land and Natural Resources - Division of Aquatic Resources.

Supplementary Table S1. Human population sizes, shark bite numbers and area of insular shelf within the 200-m isobath for each county within the State of Hawaii. Honolulu County encompasses the island of Oahu. Maui County includes the populated islands of Maui, Lanai and Molokai.

| **County** | **2015 human population*** | **Total shark bites (1995-2015)^+^** | **Grand mean annual (1995-2015) shark bites per 100,000 people** | | **Insular shelf area (km^2^)** |
| --- | --- | --- | --- | --- | --- |
| **Kauai** | 69,691 | 13 | | 1.2 | 923 |
| **Honolulu** | 984,178 | 21 | | 0.1 | 927 |
| **Maui** | 160,948 | 43 | | 1.7 | 3,641 |
| *Maui island* | *150,203* | *40* | | *1.7* |  |
| *Lanai island* | *3,539* | *2* | | *3.5* |  |
| *Molokai island* | *7,206* | *1* | | *0.9* |  |
| **Hawaii** | 191,482 | 16 | | 0.50 | 1,056 |

*Source: Hawaii Department of Business, Economic Development & Tourism (DEBDT)( http://files.hawaii.gov/dbedt/economic/databook/db2016/section01.pdf).

^+^Source: Hawaii Department of Land and Natural Resources-Division of Aquatic Resources.

Supplementary Table S2. Summary detection data for tiger sharks captured around Maui (n = 26) and Oahu (n = 15) between October 2013 and February 2015. Underlined Total Length (TL) indicates sharks above the size of sexual maturity based on reproductive data from Whitney and Crow (2007).

| **Tagging Island** | **Sex** | **Total Length (cm)** | **Date Tagged** | **Acoustic Xmitter #** | **Acoustic Detection Span (Days)** | **Total Acoustic Detection Days** | **Total Acoustic Detections** | **Satellite Xmitter #** | **Satellite Detection Span (Days)** | **Total Satellite Detections** |
| --- | --- | --- | --- | --- | --- | --- | --- | --- | --- | --- |
| Maui | M | 408 | 1/16/14 | 26895**^2^** | 449 | 32 | 102 | 81182 | 484 | 512 |
| Maui | F | 448 | 7/25/14 | 26903**^2^** | 334 | 125 | 877 | 122984**^+^** | 196 | 817 |
| Maui | F | 377 | 7/26/14 | 26891**^2^** | 306 | 69 | 308 | 132062**^+^** | 64 | 5602 |
| Maui | M | 283 | 10/19/13 | 26878**^2^** | 604 | 358 | 1695 | 133361 | 418 | 298 |
| Maui | F | 413 | 10/20/13 | 13411**^1^** | 600 | 185 | 712 | 133362 | 97 | 177 |
| Maui | F | 381 | 1/13/14 | 26888**^2^** | 517 | 264 | 1628 | 133363 | 82 | 80 |
| Maui | F | 388 | 1/13/14 | 26897**^2^** | 516 | 167 | 666 | 133364 | - | - |
| Maui | M | 323 | 1/13/14 | 26887**^2^** | 427 | 39 | 124 | 133365 | 431 | 1055 |
| Maui | F | 332 | 1/13/14 | 26892**^2^** | - | - | - | 133366 | 443 | 662 |
| Maui | F | 388 | 1/13/14 | 26896**^2^** | 503 | 182 | 767 | 133367 | 7 | 14 |
| Maui | F | 409 | 10/18/13 | 26876**^2^** | 609 | 272 | 1223 | 133368 | 79 | 17 |
| Maui | F | 375 | 10/17/13 | 26875**^2^** | 605 | 301 | 1631 | 133369 | 293 | 1537 |
| Maui | F | 432 | 10/18/13 | 26885**^2^** | - | - | - | 133370 | 55 | 48 |
| Maui | F | 373 | 10/18/13 | 26882**^2^** | 565 | 245 | 1097 | 133371 | 430 | 405 |
| Maui | F | 375 | 10/19/13 | 26879**^2^** | - | - | - | 133372 | 161 | 528 |
| Maui | F | 392 | 10/20/13 | 13407**^1^** | 613 | 347 | 1986 | 133373 | 595 | 485 |
| Maui | F | 310* | 10/19/13 | 13405**^1^** | 582 | 301 | 1244 | 144554 | 162 | 518 |
| Maui | F | 273 | 10/17/13 | 26884**^2^** | 610 | 294 | 1238 | 144555 | 93 | 277 |
| Maui | F | 401 | 12/9/14 | 30092**^3^** | 193 | 90 | 1821 | 145988 | 163 | 93 |

Supplementary Table S2 ctd.

| **Tagging Island** | **Sex** | **Total Length (cm)** | **Date Tagged** | **Acoustic Xmitter #** | **Acoustic Detection Span (Days)** | **Total Acoustic Detection Days** | **Total Acoustic Detections** | **Satellite Xmitter #** | **Satellite Detection Span (Days)** | **Total Satellite Detections** |
| --- | --- | --- | --- | --- | --- | --- | --- | --- | --- | --- |
| Maui | F | 347 | 10/20/13 | 26881**^2^** | 613 | 365 | 2235 | 145989 | 143 | 159 |
| Maui | F | 307 | 10/19/13 | 26877**^2^** | 604 | 189 | 763 |  |  |  |
| Maui | F | 246 | 10/18/13 | 26883**^2^** | 507 | 354 | 2065 |  |  |  |
| Maui | F | 282 | 1/14/14 | 26893**^2^** | 479 | 185 | 1102 |  |  |  |
| Maui | F | 379 | 12/9/14 | 26900**^2^** | 39 | 25 | 123 |  |  |  |
| Maui | F | 385 | 12/8/14 | 26901**^2^** | 117 | 86 | 425 |  |  |  |
| Maui | F | 365 | 10/20/13 | 13409**^1^** | 604 | 217 | 864 |  |  |  |
| Oahu | F | 329 | 9/19/14 | 30098**^3^** | - | - | - | 132063 | - | - |
| Oahu | M | 368 | 10/16/14 | 26898**^2^** | 152 | 25 | 71 | 137070 | 165 | 378 |
| Oahu | F | 445 | 10/16/14 | 26890**^2^** | - | - | - | 137072 | - | - |
| Oahu | F | 383 | 10/16/14 | 26880**^2^** | 236 | 37 | 166 | 137073 | 164 | 813 |
| Oahu | F | 324 | 9/19/14 | 30099**^3^** | 238 | 72 | 2318 | 137074 | 329 | 99 |
| Oahu | M | 363 | 10/8/14 | 7915**^4^** | 216 | 21 | 195 | 137077 | 280 | 199 |
| Oahu | F | 414 | 3/5/14 | 26902**^2^** | 8 | 5 | 40 | 137078 | 180 | 78 |
| Oahu | F | 379 | 9/29/14 | 30095**^3^** | 234 | 59 | 1979 | 137079 | 107 | 202 |
| Oahu | F | 255 | 2/17/15 | 18802**^5^** | 24 | 5 | 11 |  |  |  |
| Oahu | M | 203 | 2/17/15 | 38673**^5^** | - | - | - |  |  |  |
| Oahu | F | 373 | 9/17/14 | 30093**^3^** | 122 | 48 | 911 |  |  |  |
| Oahu | F | 334 | 8/23/14 | 30097**^3^** | 268 | 77 | 2320 |  |  |  |
| Oahu | M | 278 | 3/5/14 | 26889**^2^** | 399 | 32 | 130 |  |  |  |
| Oahu | M | 300 | 2/18/15 | 7910**^4^** | 99 | 26 | 674 |  |  |  |
| Oahu | F | 217 | 10/1/14 | 7913**^4^** | 245 | 33 | 529 |  |  |  |

Supplementary Table S2 ctd.

| **Tagging Island** | **Sex** | **Total Length (cm)** | **Date Tagged** | **Acoustic Xmitter #** | **Acoustic Detection Span (Days)** | **Total Acoustic Detection Days** | **Total Acoustic Detections** | **Satellite Xmitter #** | **Satellite Detection Span (Days)** | **Total Satellite Detections** |
| --- | --- | --- | --- | --- | --- | --- | --- | --- | --- | --- |
| Oahu | M | 436* | 1/8/15 |  |  |  |  |  |  |  |

| **^1^**Estimated transmitter life = 3493 days, random off time (min-max) = 130-230 seconds, temperature sensor tag | | | |
| --- | --- | --- | --- |
| **^2^**Estimated transmitter life = 3498 days, random off time (min-max) = 130-230 seconds | |  |  |
| **^3^**Estimated transmitter life = 885 days, random off time (min-max) = 20-40 seconds |  |  |  |
| **^4^**Estimated transmitter life = 707 days, random off time (min-max) = 20-40 seconds |  |  |  |
| **^5^**Estimated transmitter life = 622 days, random off time (min-max) = 30-90 seconds |  |  |  |
| *****Camera package deployed on pectoral fin |  |  |  |
| **^+^** SPLASH tag – collects and transmits depth and temperature data |  |  |  |

Supplementary Table S3. Summary metadata for “legacy” tiger sharks captured and acoustically-tagged around Oahu (n = 17), French Frigate Shoals (n = 1), Lisianski Island (n = 1) and Pearl and Hermes Atoll (n = 1) during earlier research projects (2009-2013). These sharks were all equipped with transmitters that were still active during all or part of the current Maui-focused project. Underlined Total Length (TL) indicates sharks above the size of sexual maturity at tagging based on reproductive data from Whitney and Crow (2007). Age and size estimates are based on the Hawaii tiger shark growth curve from Meyer et al. (2014).

| **Tagging Island** | **Xmitter** | **Total Length (cm) at Tagging** | **Estimated Age at Tagging (y)** | **Sex** | **Tagging Date** | **Xmitter Death Date** | **Total Maui Detection Days** | **Estimated Age (y) at First Maui Detection** | **Estimated Total Length (cm) at First Maui Detection** |
| --- | --- | --- | --- | --- | --- | --- | --- | --- | --- |
| FFS | 5659^1^ | 380 | 8.5 | F | 6/7/08 | 6/5/18 | 0 |  |  |
| Lisianski | 660^2^ | 410 |  | F | 7/14/07 | 10/30/13 | 0 |  |  |
| Oahu | 371^3^ | 333 | 4.9 | M | 2/19/08 | 12/24/14 | 0 |  |  |
| Oahu | 374^3^ | 323 | 4.4 | F | 9/10/08 | 7/16/15 | 0 |  |  |
| Oahu | 375^3^ | 276 | 3.0 | F | 2/2/07 | 12/7/13 | 0 |  |  |
| Oahu | 376^3^ | 335 | 5.0 | F | 9/27/07 | 8/1/14 | 0 |  |  |
| Oahu | 377^3^ | 430 |  | F | 9/27/07 | 8/1/14 | 0 |  |  |
| Oahu | 379^3^ | 201 | 1.5 | F | 2/4/08 | 12/9/14 | 0 |  |  |
| Oahu | 381^3^ | 312 | 4.0 | F | 9/27/07 | 8/1/14 | 0 |  |  |
| Oahu | 54782^4^ | 295 | 3.5 | M | 5/27/09 | 11/12/13 | 0 |  |  |
| Oahu | 54786^4^ | 327 | 4.6 | M | 10/6/09 | 3/24/14 | 2 | 8.8 | 382 |
| Oahu | 54790^5^ | 183 | 1.2 | F | 5/19/09 | 5/17/19 | 26 | 5.9 | 352 |
| Oahu | 54791^5^ | 256 | 2.5 | F | 2/6/09 | 2/4/19 | 2 | 8.5 | 380 |
| Oahu | 54792^5^ | 256 | 2.5 | F | 1/28/09 | 1/26/19 | 0 |  |  |
| Oahu | 54793^5^ | 287 | 3.3 | F | 2/6/09 | 2/4/19 | 53 | 8.1 | 377 |
| Oahu | 54795^5^ | 268 | 2.8 | F | 9/17/09 | 9/15/19 | 0 |  |  |
| Oahu | 54796^5^ | 291 | 3.4 | F | 11/17/09 | 11/15/19 | 0 |  |  |
| Oahu | 61971^6^ | 254 | 2.4 | F | 4/10/13 | 3/11/15 | 0 |  |  |
| Oahu | 30100^7^ | 319 | 4.3 | F | 4/22/13 | 9/24/15 | 22 | 5.8 | 350 |
| PHR | 661^8^ | 395 | 11.9 | F | 8/31/07 | 12/17/13 | 0 |  |  |

^1^Estimated transmitter life = 3650 days, random off time (min-max) = 150-300 seconds

Supplementary Table S3 ctd.

^2^Estimated transmitter life = 2300 days, random off time (min-max) = 150-300 seconds

^3^Estimated transmitter life = 2500 days, random off time (min-max) = 150-300 seconds

^4^Estimated transmitter life = 1630 days, random off time (min-max) = 30-90 seconds

^5^Estimated transmitter life = 3650 days, random off time (min-max) = 30-90 seconds, low power (V16-6L)

^6^Estimated transmitter life = 700 days, random off time (min-max) = 10-35 seconds

^7^Estimated transmitter life = 885 days, random off time (min-max) = 20-40 seconds

^8^Estimated transmitter life = 2300 days, random off time (min-max) = 150-300 seconds

Supplementary Table S4. Summary acoustic detection data for tiger sharks captured off Hawaii Island 2003-2004. All sharks were equipped with acoustic transmitters with anticipated battery lives of 732 days, and random off time (min-max) = 10-35 seconds. Underlined Total Length (TL) indicates sharks above the size of sexual maturity at tagging based on reproductive data from Whitney and Crow (2007).

| **Sex** | **Total Length (cm)** | **Date Tagged** | **Acoustic Xmitter #** | **Acoustic Detection Span (Days)** | **Total Acoustic Detection Days** | **Total Acoustic Detections** |
| --- | --- | --- | --- | --- | --- | --- |
| F | 219 | 12/3/03 | 304 | 72 | 19 | 51 |
| F | 355 | 12/4/03 | 305 | 497 | 19 | 53 |
| F | 375 | 12/3/04 | 313 | 792 | 46 | 648 |
| F | 383 | 12/1/04 | 317 | 924 | 140 | 1364 |
| F | 413 | 12/1/04 | 306 | 495 | 47 | 560 |
| F | 439 | 6/26/04 | 316 | 820 | 70 | 856 |
| F | 449 | 12/3/03 | 303 | 768 | 66 | 208 |
| F | 460 | 12/4/03 | 301 | 101 | 49 | 421 |
| M | 181 | 6/27/04 | 309 | 779 | 72 | 780 |
| M | 218 | 6/27/04 | 311 | 0 | 1 | 2 |
| M | 279 | 6/27/04 | 315 | 42 | 15 | 186 |

Supplementary Table S5. Summary acoustic detection data for tiger sharks captured at French Frigate Shoals atoll in 2009. All sharks were equipped with acoustic transmitters with anticipated battery lives of 700 days, and random off time (min-max) = 10-35 seconds. Underlined Total Length (TL) indicates sharks above the size of sexual maturity at tagging based on reproductive data from Whitney and Crow (2007).

| **Sex** | **Total Length (cm)** | **Date Tagged** | **Acoustic Xmitter #** | **Acoustic Detection Span (Days)** | **Total Acoustic Detection Days** | **Total Acoustic Detections** |
| --- | --- | --- | --- | --- | --- | --- |
| F | 450 | 5/27/09 | 55222 | 559 | 17 | 131 |
| F | 444 | 6/30/09 | 55887 | 220 | 33 | 275 |
| F | 443 | 6/7/09 | 55193 | 703 | 138 | 1825 |
| F | 440 | 6/14/09 | 55220 | 700 | 88 | 1076 |
| F | 437 | 5/23/09 | 55901 | 501 | 7 | 10 |
| F | 434 | 5/11/09 | 55924 | 674 | 91 | 909 |
| F | 432 | 7/21/09 | 55872 | 2 | 2 | 27 |
| F | 431 | 6/11/09 | 55898 | 666 | 28 | 154 |
| F | 431 | 7/30/09 | 59502 | 0 | 1 | 2 |
| F | 425 | 7/5/09 | 59515 | 321 | 15 | 120 |
| F | 420 | 7/17/09 | 59508 | 771 | 346 | 4697 |
| F | 418 | 5/24/09 | 55914 | 897 | 103 | 1192 |
| F | 418 | 7/4/09 | 59516 | 361 | 18 | 134 |
| F | 416 | 6/29/09 | 55886 | 569 | 44 | 180 |
| F | 410 | 7/16/09 | 55870 | 60 | 12 | 90 |
| F | 409 | 6/7/09 | 55191 | 492 | 15 | 140 |
| F | 408 | 5/10/09 | 55921 | 423 | 19 | 129 |
| F | 406 | 7/6/09 | 55869 | 267 | 18 | 114 |
| F | 405 | 6/15/09 | 55236 | 659 | 13 | 70 |
| F | 395 | 7/4/09 | 59519 | 690 | 172 | 1877 |
| F | 395 | 7/2/09 | 59523 | 711 | 45 | 462 |
| F | 393 | 6/29/09 | 55228 | 661 | 51 | 595 |
| F | 372 | 6/7/09 | 55192 | 272 | 55 | 809 |
| F | 371 | 7/6/09 | 59513 | 700 | 78 | 1919 |
| F | 370 | 7/27/09 | 55876 | 383 | 57 | 617 |
| F | 360 | 5/20/09 | 55893 | 702 | 15 | 237 |
| F | 350 | 6/12/09 | 59535 | 769 | 32 | 241 |
| F | 320 | 5/21/09 | 55895 | 594 | 330 | 4203 |
| F | 302 | 5/26/09 | 55221 | 688 | 284 | 3237 |
| F | 272 | 6/15/09 | 55227 | 949 | 164 | 2359 |
| F | 261 | 7/1/09 | 55889 | 193 | 10 | 37 |
|  |  |  |  |  |  | /ctd. |
| Supplementary Table S5 ctd. | |  |  |  |  |  |
| **Sex** | **Total Length (cm)** | **Date Tagged** | **Acoustic Xmitter #** | **Acoustic Detection Span (Days)** | **Total Acoustic Detection Days** | **Total Acoustic Detections** |
| M | 406 | 6/4/09 | 55223 | 684 | 46 | 579 |
| M | 403 | 7/6/09 | 59525 | 733 | 30 | 274 |
| M | 363 | 7/2/09 | 55207 | 622 | 25 | 141 |
| M | 347 | 5/24/09 | 55907 | 792 | 101 | 1123 |
| M | 337 | 6/4/09 | 55218 | 696 | 157 | 1178 |
| M | 328 | 6/15/09 | 55229 | 693 | 92 | 2009 |
| M | 291 | 5/23/09 | 55912 | 691 | 74 | 842 |
| M | 284 | 6/14/09 | 59533 | 20 | 2 | 23 |

**
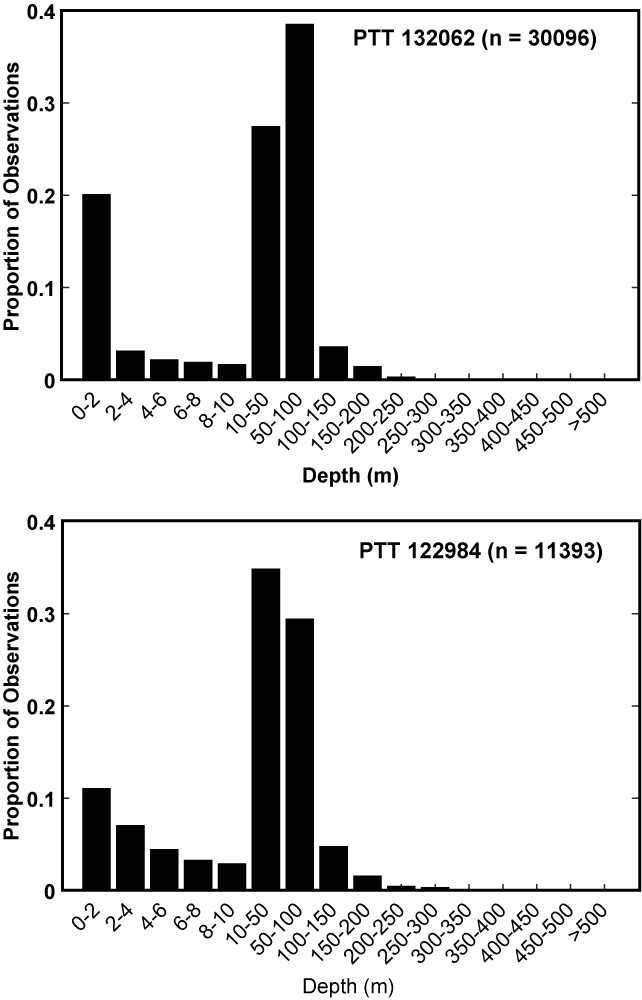
**

Supplementary Figure S3. Swimming depth frequency distribution from tiger shark vertical profiles collected by SPLASH tags. Sample sizes (n) indicate the number of depth measurements taken by each tag. N.B. Different bin sizes are used on the horizontal axis to provide a higher resolution view of tiger shark use of surface (0-10m) waters.

**
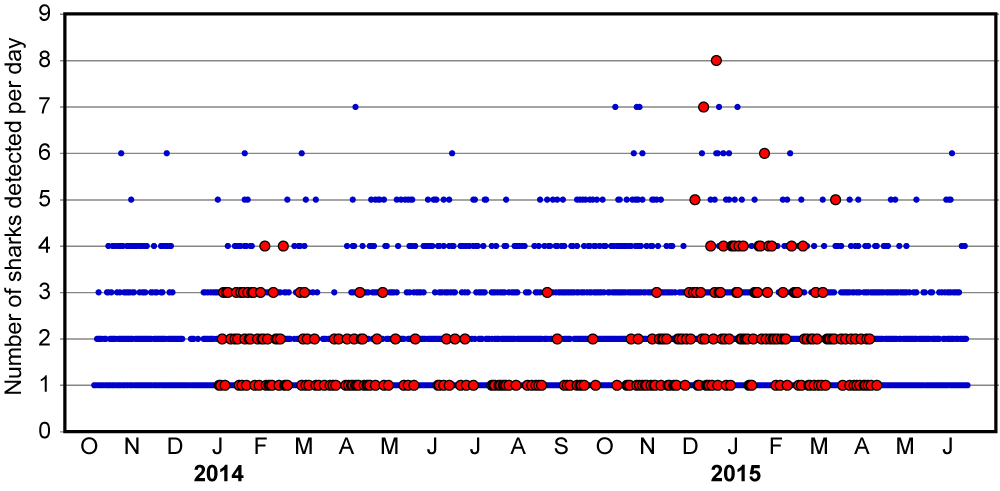
**

Supplementary Figure S4. Scatter plot illustrating detections of multiple sharks on the same day at Maui receiver sites. Blue points: number of sharks detected per day (y axis) at all Maui receiver sites except Olowalu. Red points: number of sharks detected per day (y axis) at Olowalu. Note how detections of multiple individuals peak during tiger shark mating season (Jan-Mar) at Olowalu.

**
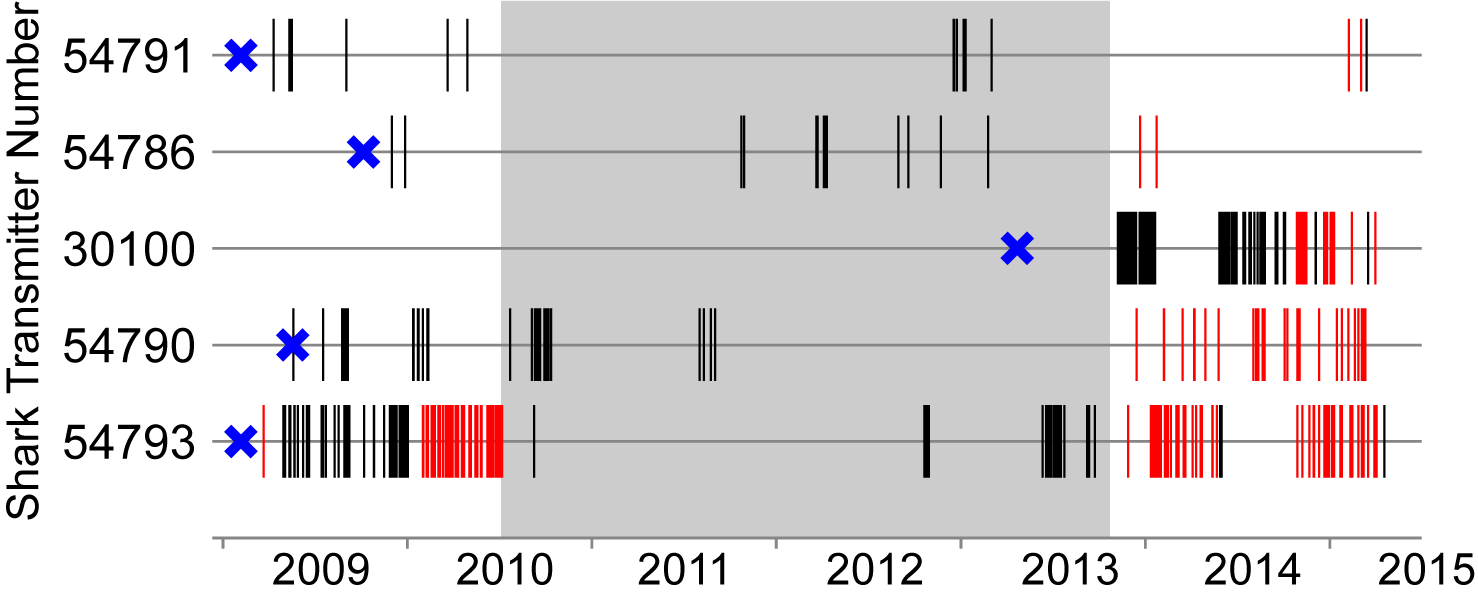
**

Supplementary Figure S5. Abacus plot showing multi-year detections of five ‘legacy’ tiger sharks tagged on Oahu (2009-2013) and subsequently detected around Oahu (black vertical lines) and Maui (red vertical lines). Shaded section indicates period of no acoustic monitoring around Maui. Blue crosses indicate capture and tagging dates.

Supplementary Table S6. Results of one-way analysis of variance (ANOVA) examining the influence of shark tagging island on overall average Site Fidelity Index (SFI) and average maximum SFI values. Note that mean SFI data exhibited unequal variance among islands. This was accounted for by using a Welch's ANOVA (with post-hoc Games-Howell test). Maximum SFI was evaluated using a one-way ANOVA (with post hoc Tukey's HSD test). s.e., standard error.

|  |  | **n** | **Mean** | **s.e.** |  |  |  |
| --- | --- | --- | --- | --- | --- | --- | --- |
| **Mean SFI** | FFS | 38 | 1.31^a^ | 0.22 |  |  |  |
|  | Oahu | 12 | 3.72^b^ | 0.91 |  |  |  |
|  | Maui | 23 | 6.34^b^ | 0.74 |  |  |  |
|  | Hawaii | 11 | 0.80^a^ | 0.09 |  |  |  |
|  |  |  |  |  |  |  |  |
| **Max SFI** | FFS | 38 | 3.70^a^ | 0.68 |  |  |  |
|  | Oahu | 12 | 11.68^b^ | 2.67 |  |  |  |
|  | Maui | 23 | 19.13^b^ | 2.05 |  |  |  |
|  | Hawaii | 11 | 2.78^a^ | 0.49 |  |  |  |

Islands without a shared letter were significantly different (Games-Howell test and Tukey’s HSD test, df = 3, P < 0.05).

|  |  | **df*_among_*** | **df*_within_*** | ***F*** | **P-value** |
| --- | --- | --- | --- | --- | --- |
| **Mean SFI*** |  | 3 | 33.4 | 42.4 | < 0.0001 |
|  |  |  |  |  |  |
| **Max SFI** |  | 3 | 80 | 27.6 | < 0.0001 |
|  |  |  |  |  |  |

*Welch’s ANOVA

Supplementary Table S7. Ranked generalized linear mixed-effects models of spatial and biological effects on probability of tiger shark presence at acoustically monitored sites around the islands of Maui and Oahu. Models with a ΔAIC*_c_* < 2 from the model with the lowest AIC*_c_* are shown. Values in bold indicate the most parsimonious model. n = 1066.

| Model | df | logLik | AIC*_c_* | ΔAIC*_c_* | *w* | %DE |
| --- | --- | --- | --- | --- | --- | --- |
| Receiver Depth + DistTagRec + Island + Sex + Receiver Depth × Sex | 8 | -298.597 | 613.3 | 0.00 | 0.116 | 42.31 |
| DistTagRec + Island | 5 | -301.734 | 613.5 | 0.19 | 0.105 | 41.71 |
| **DistTagRec** | **4** | **-303.037** | **614.1** | **0.78** | **0.078** | **41.45** |
| Receiver Depth + DistTagRec + Sex + Receiver Depth × Sex | 7 | -300.032 | 614.2 | 0.84 | 0.076 | 42.03 |
| DistTagRec + Island + Sex | 6 | -301.335 | 614.7 | 1.42 | 0.057 | 41.78 |
| Receiver Depth + DistTagRec + Island + Sex + Receiver Depth × DistTagRec + Receiver Depth × Sex | 9 | -298.455 | 615.1 | 1.75 | 0.048 | 42.34 |
| Receiver Depth + DistTagRec + Island + Sex + TL + Receiver Depth × Sex | 9 | -298.577 | 615.3 | 1.99 | 0.043 | 42.32 |

df, degrees of freedom; logLik, maximum log-likelihood; %DE, percent deviance explained; DistTagRec, distance between shark tagging site and receiver location

Supplementary Table S8. Ranked generalized linear mixed-effects models of spatial and biological effects on probability of tiger shark presence at acoustically monitored sites around the islands of Maui, Oahu, Hawaii Island and French Frigate Shoals atoll. Models with a ΔAIC*_c_* < 2 from the model with the lowest AIC*_c_* are shown. Values in bold indicate the most parsimonious model. n = 2249.

| Model | df | logLik | AIC*_c_* | ΔAIC*_c_* | *w* | %DE |
| --- | --- | --- | --- | --- | --- | --- |
| **Receiver Depth + DistTagRec + Island + Receiver Depth × DistTagRec** | **9** | **-745.272** | **1508.6** | **0.00** | **0.294** | **23.66** |
| Receiver Depth + DistTagRec + Island + Sex + Receiver Depth × DistTagRec | 10 | -745.002 | 1510.1 | 1.48 | 0.140 | 23.68 |
| Receiver Depth + DistTagRec + Island + TL + Receiver Depth × DistTagRec | 10 | -745.231 | 1510.6 | 1.94 | 0.112 | 23.66 |

df, degrees of freedom; logLik, maximum log-likelihood; %DE, percent deviance explained; DistTagRec, distance between shark tagging site and receiver location


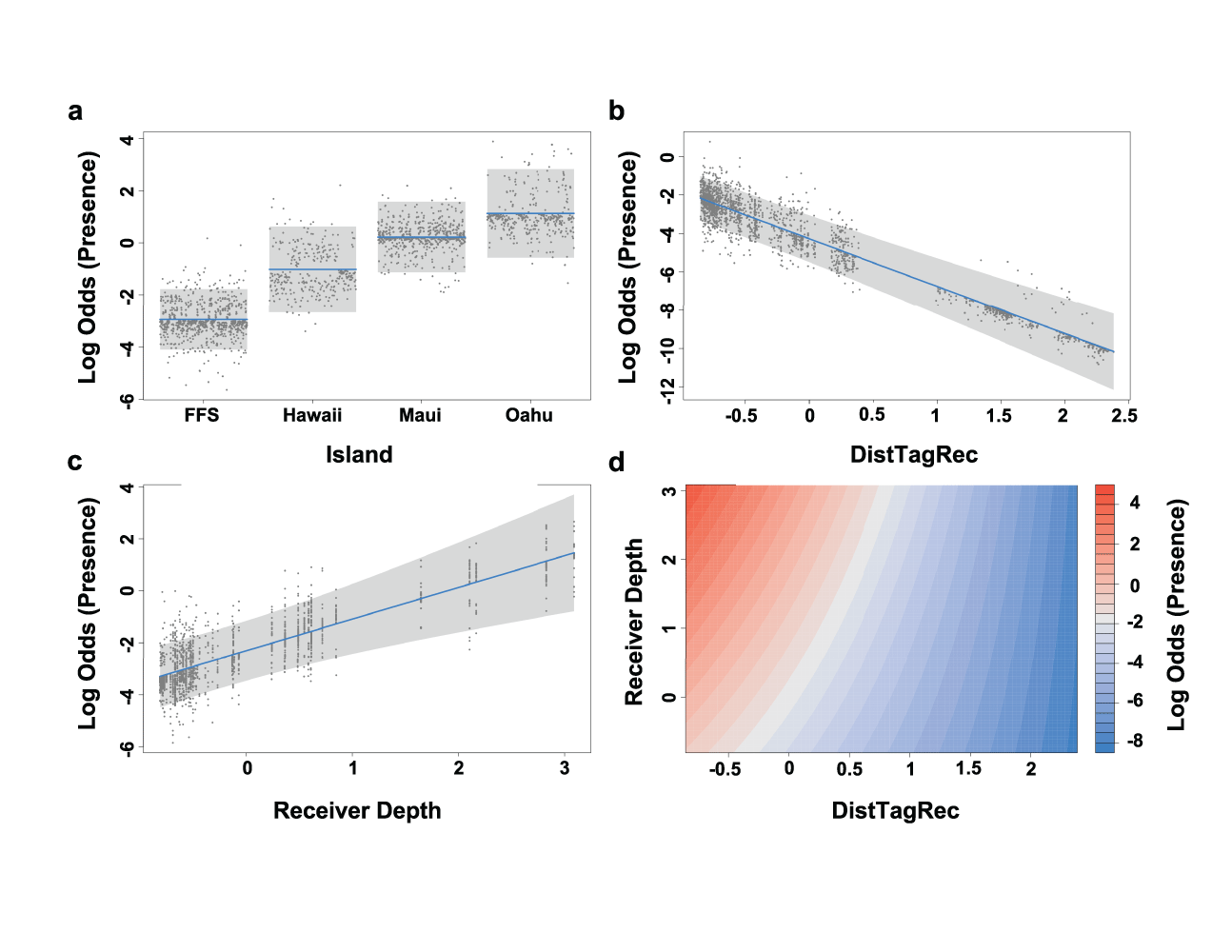


Supplementary Figure S6. Modelled effects on the daily presence of tiger sharks at receiver locations around Maui, Oahu, Hawaii Island, and French Frigate Shoals atoll. Estimated responses (lines) of (a) tagging island, (b) distance between shark tagging location and receiver site, and (c) receiver depth on the daily presence of tiger sharks from the final GLMM. Shaded areas represent 95% confidence limits and points are partial residuals. Vertical axes are log odds on the scale of the linear predictor. (d) Contour plot of the interaction between receiver depth and the distance between shark tagging location and receiver site. Note distance and depth values are mean centered and scaled.

Supplementary Table S9. Number and depth of receivers deployed around French Frigate Shoals (FFS), Oahu, Maui and Hawaii Island.

| **Island** | **No. of**  **Receivers** | **Receiver Depth (m)** | | |
| --- | --- | --- | --- | --- |
|  |  | **Minimum** | **Maximum** | **Mean** |
| FFS | 24 | 0.9 | 45.7 | 8.9 |
| Oahu | 12 | 11.9 | 195.7 | 87.2 |
| Maui | 15 | 7.9 | 78.6 | 37.8 |
| Hawaii | 36 | 3.0 | 37.5 | 18.6 |

Supplementary Table S10. Ranked generalized additive mixed models of spatial and biological effects on tiger shark site fidelity at acoustically monitored sites around the islands of Maui and Oahu. Models with a ΔAIC*_c_* < 2 from the model with the lowest AIC*_c_* are shown. s() denotes a smoother term. Values in bold indicate the most parsimonious model. n = 321.

| Model | df | logLik | AIC*_c_* | ΔAIC*_c_* | *w* | %DE |
| --- | --- | --- | --- | --- | --- | --- |
| **Island + Sex + s(DistTagRec)** | **7** | **-2745.064** | **5504.5** | **0.00** | **0.472** | **48.62** |

df, degrees of freedom; logLik, maximum log-likelihood; %DE, percent deviance explained; DistTagRec, distance between shark tagging site and receiver location

Supplementary Table S11. Ranked generalized additive mixed models of spatial and biological effects on tiger shark site fidelity at acoustically monitored sites around the islands of Maui, Oahu, Hawaii Island and French Frigate Shoals atoll. Models with a ΔAIC*_c_* < 2 from the model with the lowest AIC*_c_* are shown. s() denotes a smoother term. Values in bold indicate the most parsimonious model. n = 723.

| Model | df | logLik | AIC*_c_* | ΔAIC*_c_* | *w* | %DE |
| --- | --- | --- | --- | --- | --- | --- |
| **Island + s(Receiver Depth) + s(DistTagRec)** | **10** | **-4907.427** | **9835.2** | **0.00** | **0.462** | **47.60** |
| Island + Sex + s(Receiver Depth) + s(DistTagRec) | 11 | -4907.080 | 9836.5 | 1.37 | 0.233 | 48.11 |

df, degrees of freedom; logLik, maximum log-likelihood; %DE, percent deviance explained; DistTagRec, distance between shark tagging site and receiver location


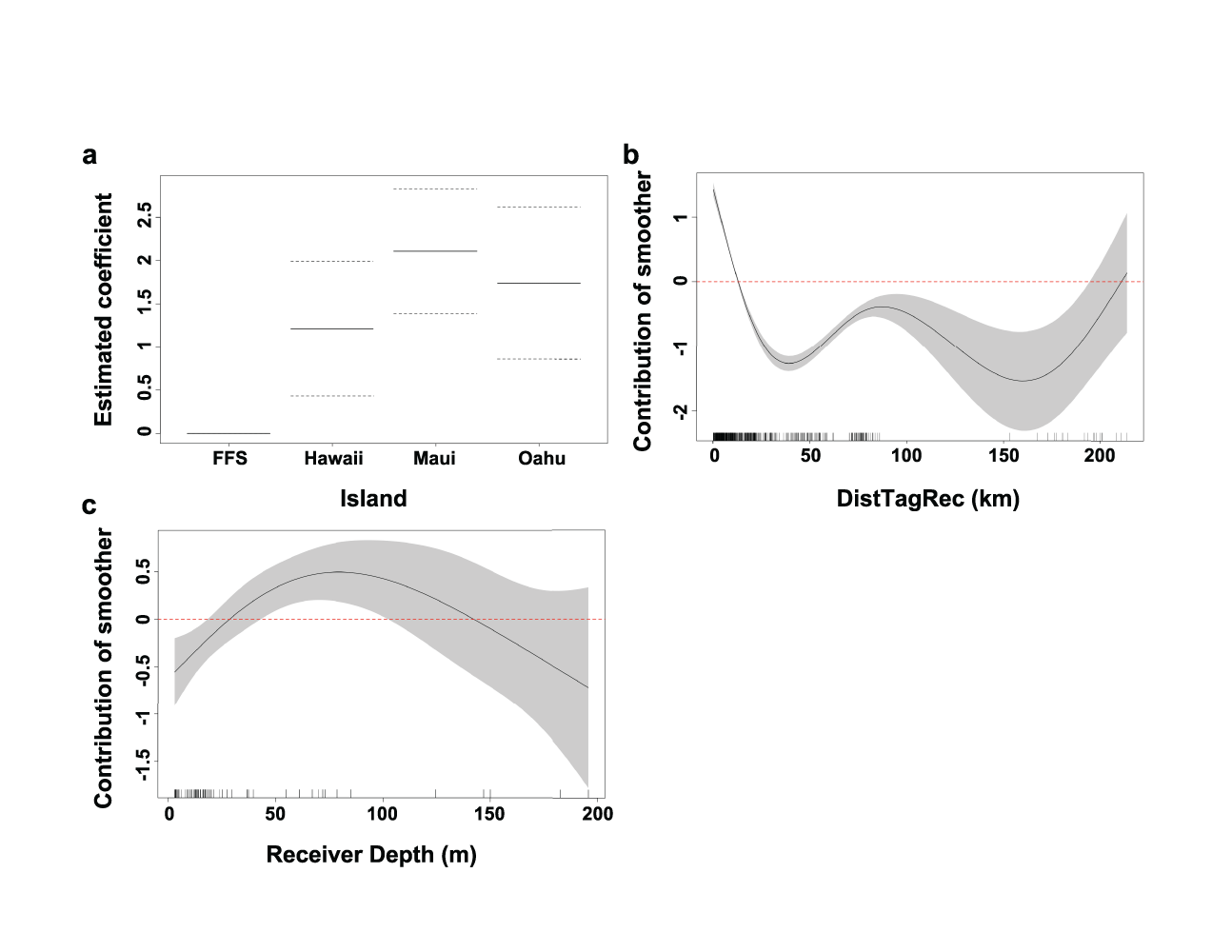


Supplementary Figure S7. Modelled effects on the site fidelity index (excluding zeros) of tiger sharks at receiver locations around Maui, Oahu, Hawaii Island, and French Frigate Shoals atoll. (a) Estimated partial coefficients for the parametric explanatory variable of tagging island on site fidelity index from the final GAMM. Base level of French Frigate Shoals atoll is centered and dashed lines represent 95% confidence limits. (b-c) Estimated response curves (black solid line) of component smooth functions. Shaded areas represent 95% confidence limits of uncertainty in the centered smooth. Vertical axes are partial responses (estimated, centered smooth functions) on the scale of the linear predictor. Ticks on x-axis denote values for which there are data. Positive values on y-axis (above red dashed line) indicate increased site fidelity index.

Supplementary Table S12. Results of tests examining the influence of shark tagging island on overall mean and average maximum visit frequency (visits per day) to receiver sites. An unequal variance F-test (Welch's ANOVA with post-hoc Games-Howell test) was used to compare overall mean visit frequency to receiver sites among tagging islands. Due to non-normality of transformed residuals, a non-parametric Kruskal-Wallis test (with Wilcoxon rank-sum test) was used to compare average maximum visit frequency among tagging islands. s.e., standard error.

|  |  | **n** | **Mean** | **s.e.** |  |
| --- | --- | --- | --- | --- | --- |
| **Mean Daily Visit Frequency** | FFS | 38 | 0.016^a^ | 0.003 |  |
|  | Oahu | 12 | 0.049^b^ | 0.012 |  |
|  | Maui | 23 | 0.078^b^ | 0.010 |  |
|  | Hawaii | 11 | 0.011^a^ | 0.001 |  |
|  |  |  |  |  |  |
| **Max Daily Visit Frequency** | FFS | 38 | 3.87^a^ | 0.70 |  |
|  | Oahu | 12 | 3.58^ab^ | 0.63 |  |
|  | Maui | 23 | 4.57^b^ | 0.38 |  |
|  | Hawaii | 11 | 3.18^a^ | 0.62 |  |

Islands without a shared letter were significantly different (Games-Howell test and Wilcoxon rank-sum test, df = 3, P < 0.05).

|  |  | **df*_among_*** | **df*_within_*** | ***F*** | **P-value** |
| --- | --- | --- | --- | --- | --- |
| **Mean Daily Visit Frequency** |  | 3 | 32.8 | 37.7 | < 0.0001 |
|  |  |  |  |  |  |
|  |  |  |  |  |  |

|  |  | **df** |  | ***H*** | **P-value** |
| --- | --- | --- | --- | --- | --- |
|  |  |  |  |  |  |
| **Max Daily Visit Frequency*** |  | 3 |  | 8.54 | 0.0361 |
| ***** Kruskal-Wallis test |  |  |  |  |  |

Supplementary Table S13. Results of Welch's ANOVA (with post-hoc Games-Howell test) examining the influence of shark tagging island on overall mean and average maximum visit duration (min) to receiver sites. s.e., standard error.

|  |  | **n** | **Mean** | **s.e.** |  |
| --- | --- | --- | --- | --- | --- |
| **Mean Visit Duration (min)** | FFS | 38 | 13.1^a^ | 0.4 |  |
|  | Oahu | 12 | 19.1^b^ | 1.7 |  |
|  | Maui | 23 | 14.3^b^ | 0.3 |  |
|  | Hawaii | 11 | 9.5^c^ | 0.3 |  |
|  |  |  |  |  |  |
| **Max Visit Duration (min)** | FFS | 38 | 67.7^a^ | 10.3 |  |
|  | Oahu | 12 | 68.1^ab^ | 11.8 |  |
|  | Maui | 23 | 84.4^b^ | 7.1 |  |
|  | Hawaii | 11 | 35.0^a^ | 9.0 |  |

Islands without a shared letter were significantly different (Games-Howell test, df = 3, P < 0.05).

|  |  | **df*_among_*** | **df*_within_*** | ***F*** | **P-value** |
| --- | --- | --- | --- | --- | --- |
| **Mean Visit Duration (min)** |  | 3 | 27.4 | 34.6 | < 0.0001 |
|  |  |  |  |  |  |
| **Max Visit Duration (min)** |  | 3 | 28.6 | 8.4 | 0.0004 |
|  |  |  |  |  |  |

Supplementary Table S14. Ranked generalized additive mixed models of spatial and biological effects on tiger shark mean visit duration at acoustically monitored sites around the islands of Maui and Oahu. Models with a ΔAIC*_c_* < 2 from the model with the lowest AIC*_c_* are shown. s() denotes a smoother term. Values in bold indicate the most parsimonious model. n = 321.

| Model | df | logLik | AIC*_c_* | ΔAIC*_c_* | *w* | %DE |
| --- | --- | --- | --- | --- | --- | --- |
| **Sex + s(Receiver Depth)** | **7** | **-923.719** | **1861.8** | **0.00** | **0.379** | **34.30** |
| Island + Sex + s(Receiver Depth) | 8 | -923.235 | 1862.9 | 1.14 | 0.215 | 33.25 |

df, degrees of freedom; logLik, maximum log-likelihood; %DE, percent deviance explained

Supplementary Table S15. Ranked generalized additive mixed models of spatial and biological effects on tiger shark mean visit duration at acoustically monitored sites around the islands of Maui, Oahu, Hawaii Island and French Frigate Shoals atoll. Models with a ΔAIC*_c_* < 2 from the model with the lowest AIC*_c_* are shown. s() denotes a smoother term. Values in bold indicate the most parsimonious model. n = 722.

| Model | df | logLik | AIC*_c_* | ΔAIC*_c_* | *w* | %DE |
| --- | --- | --- | --- | --- | --- | --- |
| Island + s(Receiver Depth) | 9 | -1939.547 | 3897.3 | 0.00 | 0.236 | 33.07 |
| **s(Receiver Depth)** | **6** | **-1942.668** | **3897.5** | **0.11** | **0.224** | **26.38** |
| s(Receiver Depth) + s(DistTagRec) | 8 | -1941.419 | 3899.0 | 1.69 | 0.101 | 27.85 |
| Sex + s(Receiver Depth) | 7 | -1942.454 | 3899.1 | 1.72 | 0.100 | 26.94 |
| Island + Sex + s(Receiver Depth) | 10 | -1939.418 | 3899.1 | 1.80 | 0.096 | 33.33 |

df, degrees of freedom; logLik, maximum log-likelihood; %DE, percent deviance explained; DistTagRec, distance between shark tagging site and receiver location


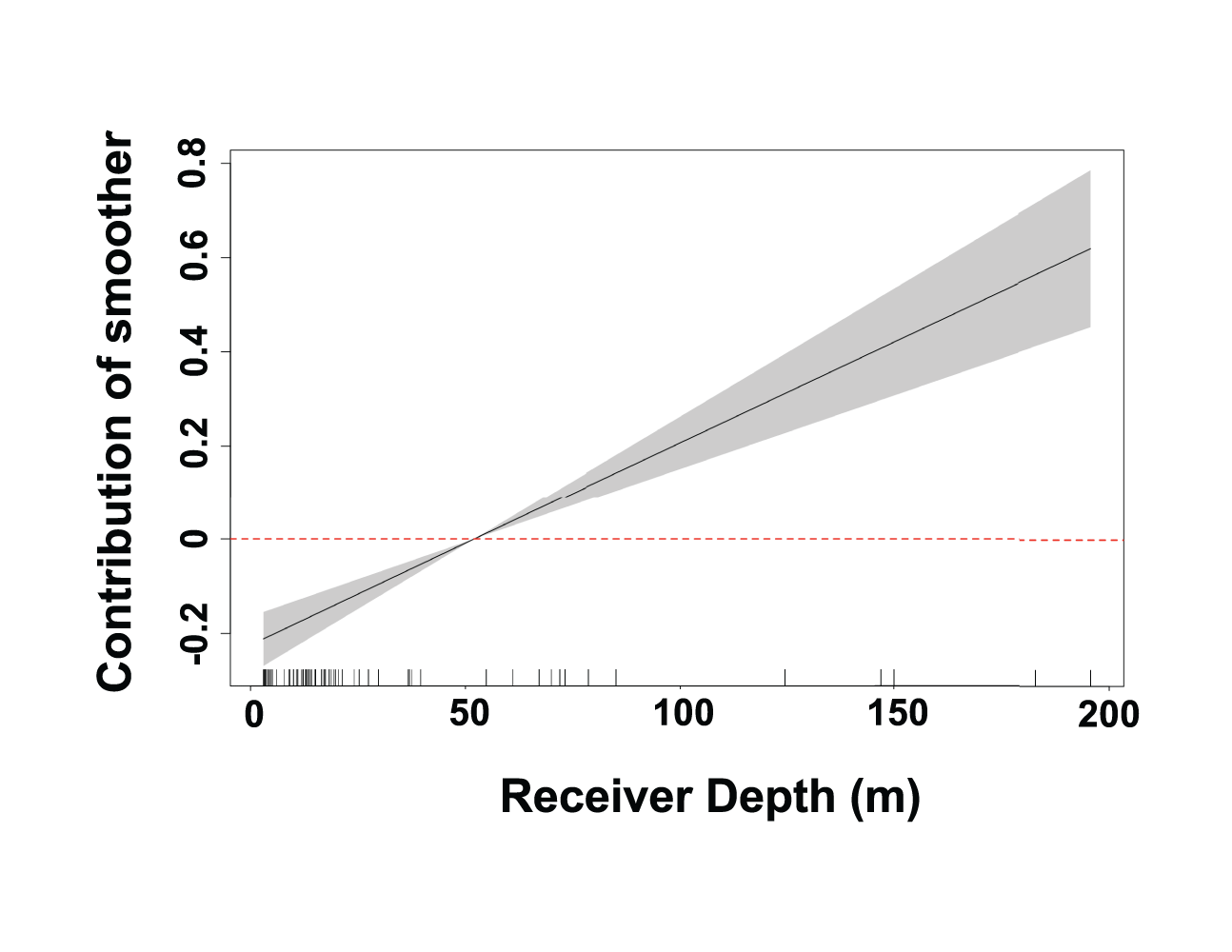


Supplementary Figure S8. Modelled effects on the mean visit duration of tiger sharks at receiver locations around Maui, Oahu, Hawaii Island, and French Frigate Shoals atoll. Estimated response curve (black solid line) of component smooth function. Shaded area represents 95% confidence limits of uncertainty in the centered smooth. Vertical axis is the partial response (estimated, centered smooth function) on the scale of the linear predictor. Ticks on x-axis denote values for which there are data. Positive values on y-axis (above red dashed line) indicate increased mean visit duration.

**
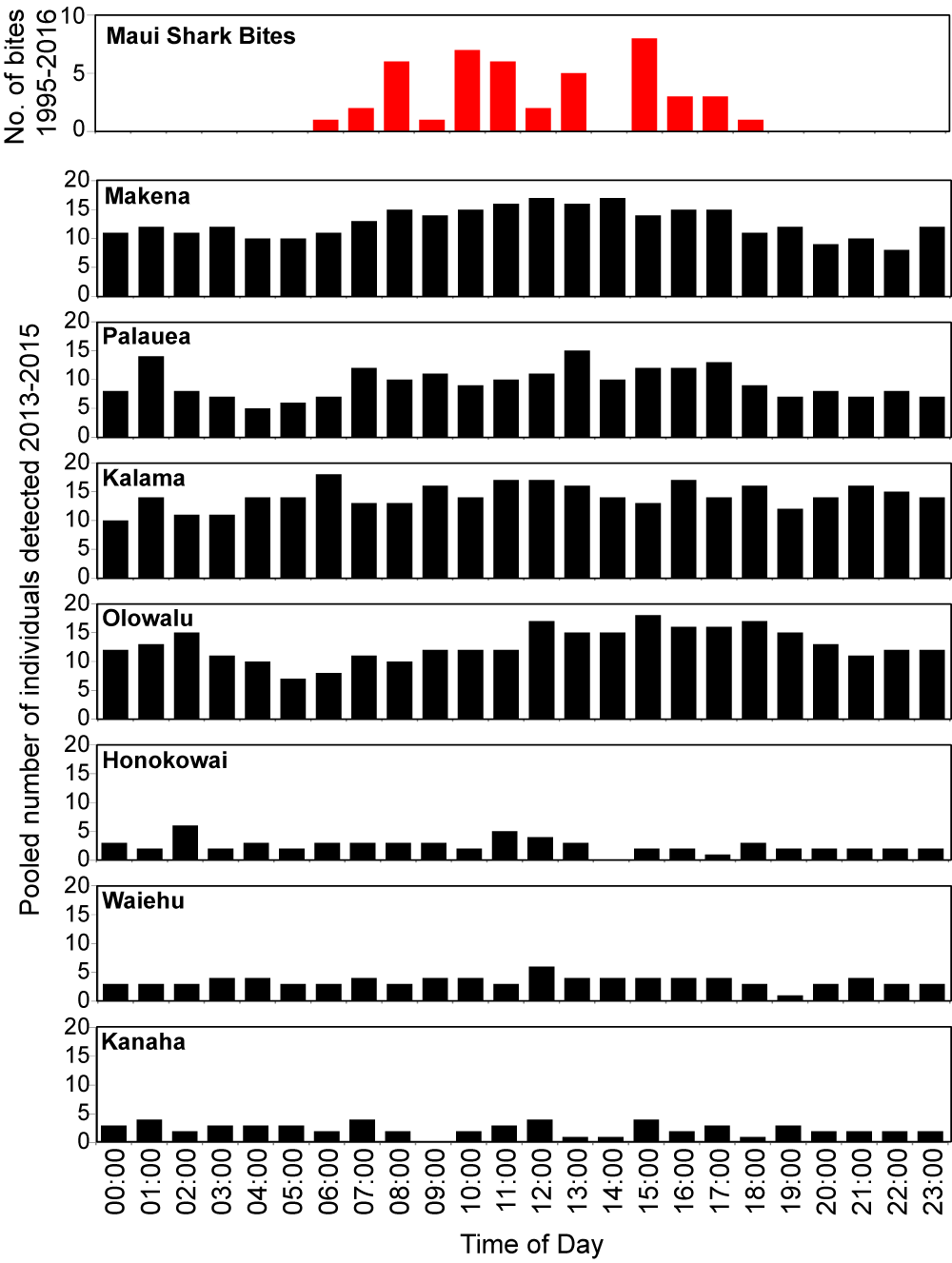
**

Supplementary Fig. S9. Diel patterns of shark bite incidents around Maui 1995-2016 (top in red) and pooled numbers of tagged tiger sharks detected at ocean recreation sites around Maui 2013-2015 (black).

**
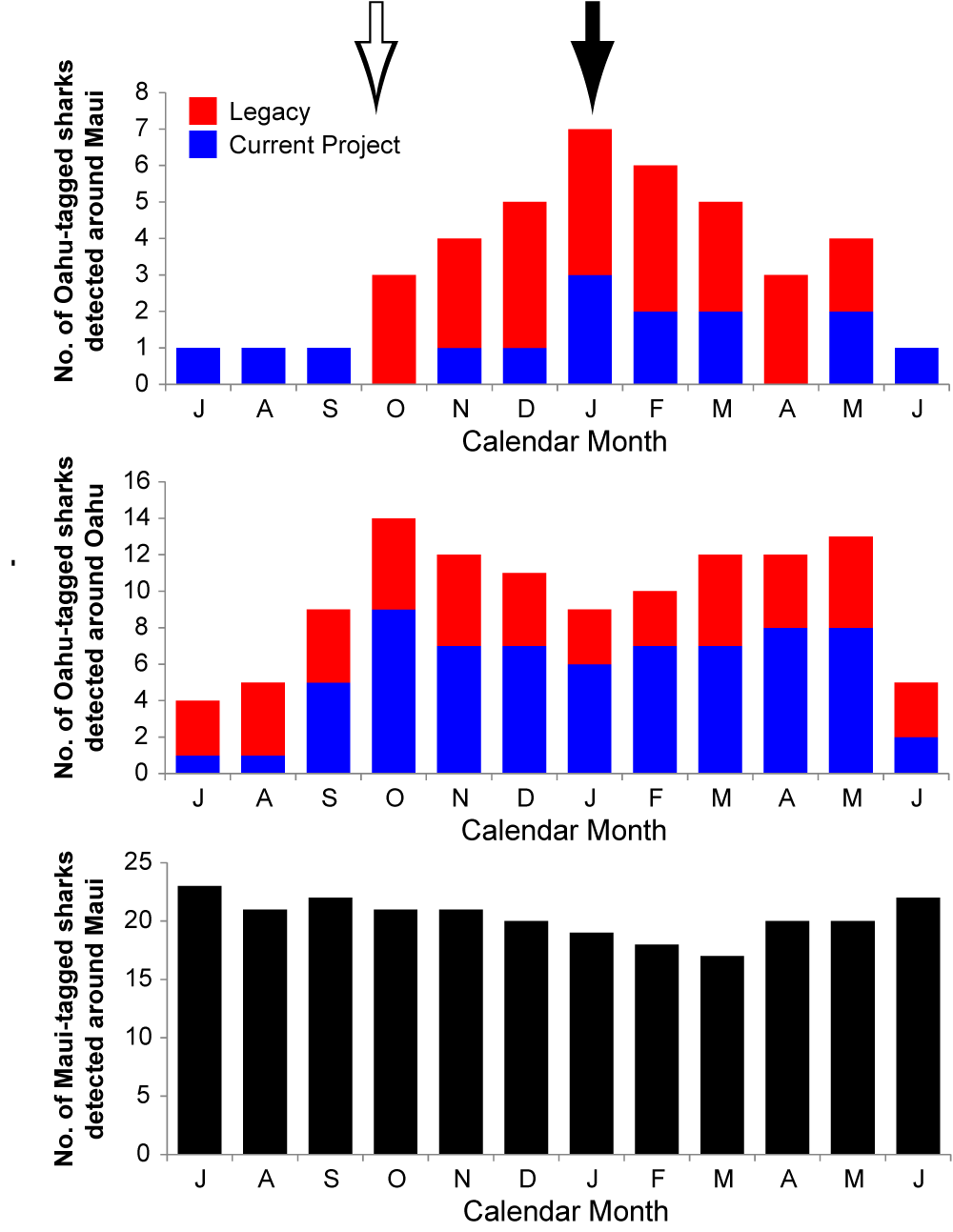
**

Supplementary Fig. S10. Monthly number of Oahu-tagged tiger sharks detected around Maui. Blue: sharks tagged during current 2013-2015 project. Red: ‘legacy’ sharks tagged around Oahu 2009-2013. Open arrow: peak tiger shark pupping season (Whitney and Crow 2007). Closed arrow: peak tiger shark mating season (Whitney and Crow 2007).


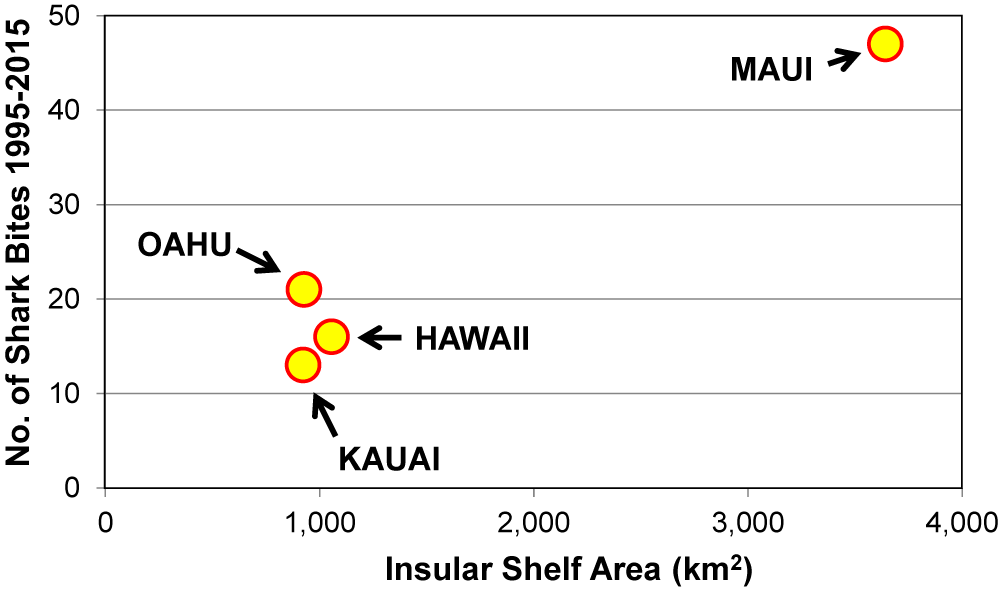


Supplementary Figure S11. Total number of shark bites 1995-2015 versus the insular shelf area (km^2^) of Hawaii, Maui, Oahu and Kauai counties.

**
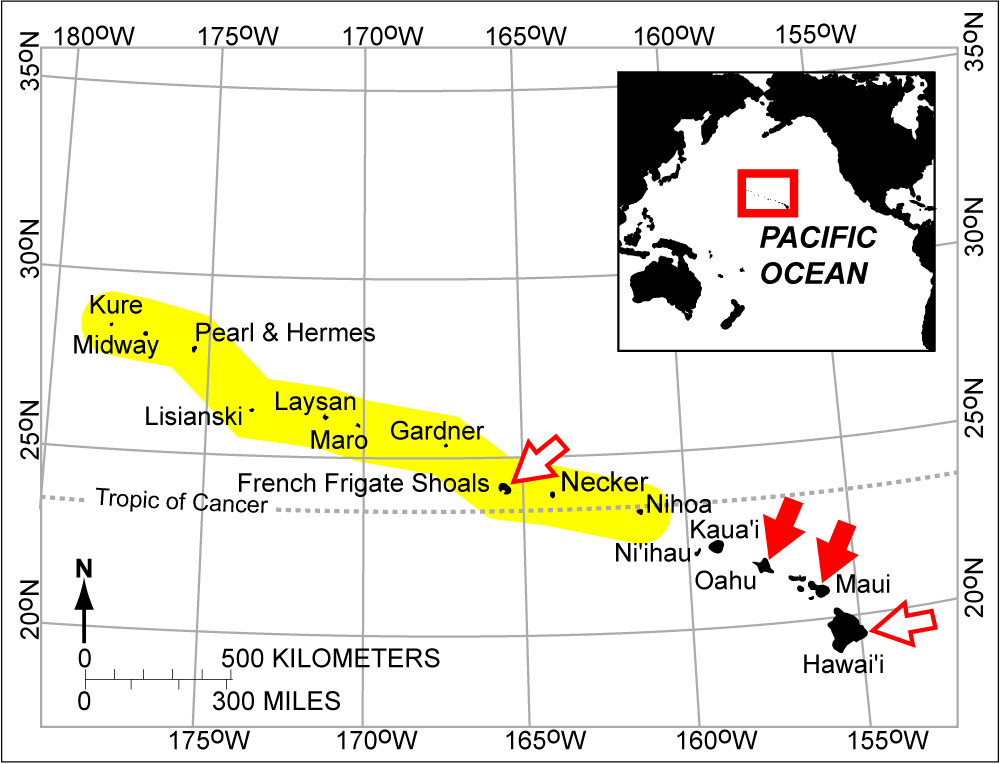
**

Supplementary Fig. S12. Hawaiian Archipelago showing locations of islands around which tiger shark tracking was conducted during the current (closed arrows) and previous (open arrows) studies. Yellow shaded area indicates the Papahānaumokuākea Marine National Monument (North West Hawaiian Islands). Inset: Location of the Hawaiian Archipelago (red box) in the north Pacific. Map was created using R software v.3.1.2 (R: A Language and Environment for Statistical Computing, R Core Team, R Foundation for Statistical Computing, Vienna, Austria (2014) https://www.R-project.org).


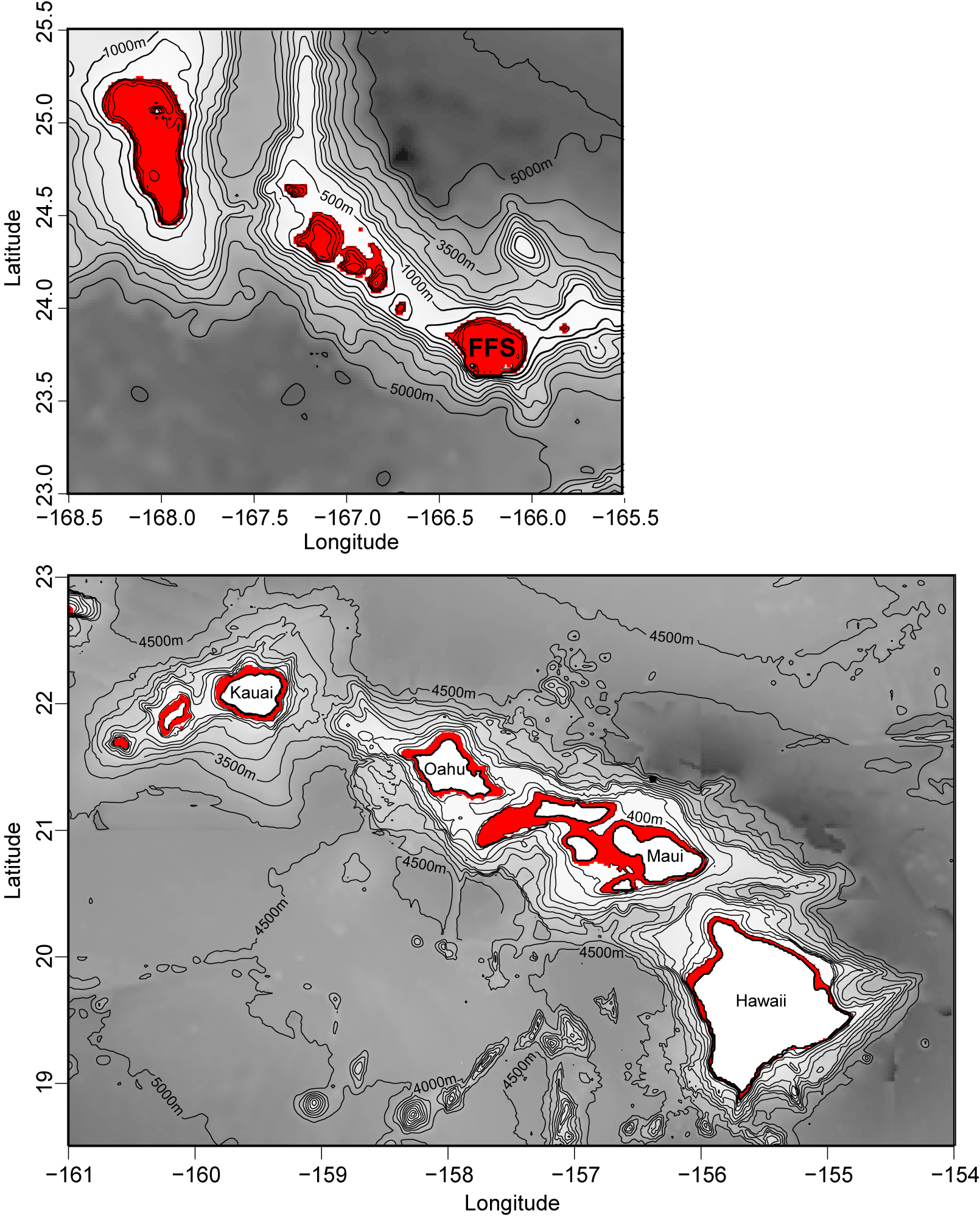


Supplementary Fig. S13. Bathymetry of French Frigate Shoals atoll (FFS) and adjacent submerged banks (top), and (bottom) the Main Hawaiian Islands highlighting the insular shelf between depths of zero and 200 m (red shaded area). Maps were created using R software v.3.1.2 (R: A Language and Environment for Statistical Computing, R Core Team, R Foundation for Statistical Computing, Vienna, Austria (2014) https://www.R-project.org).


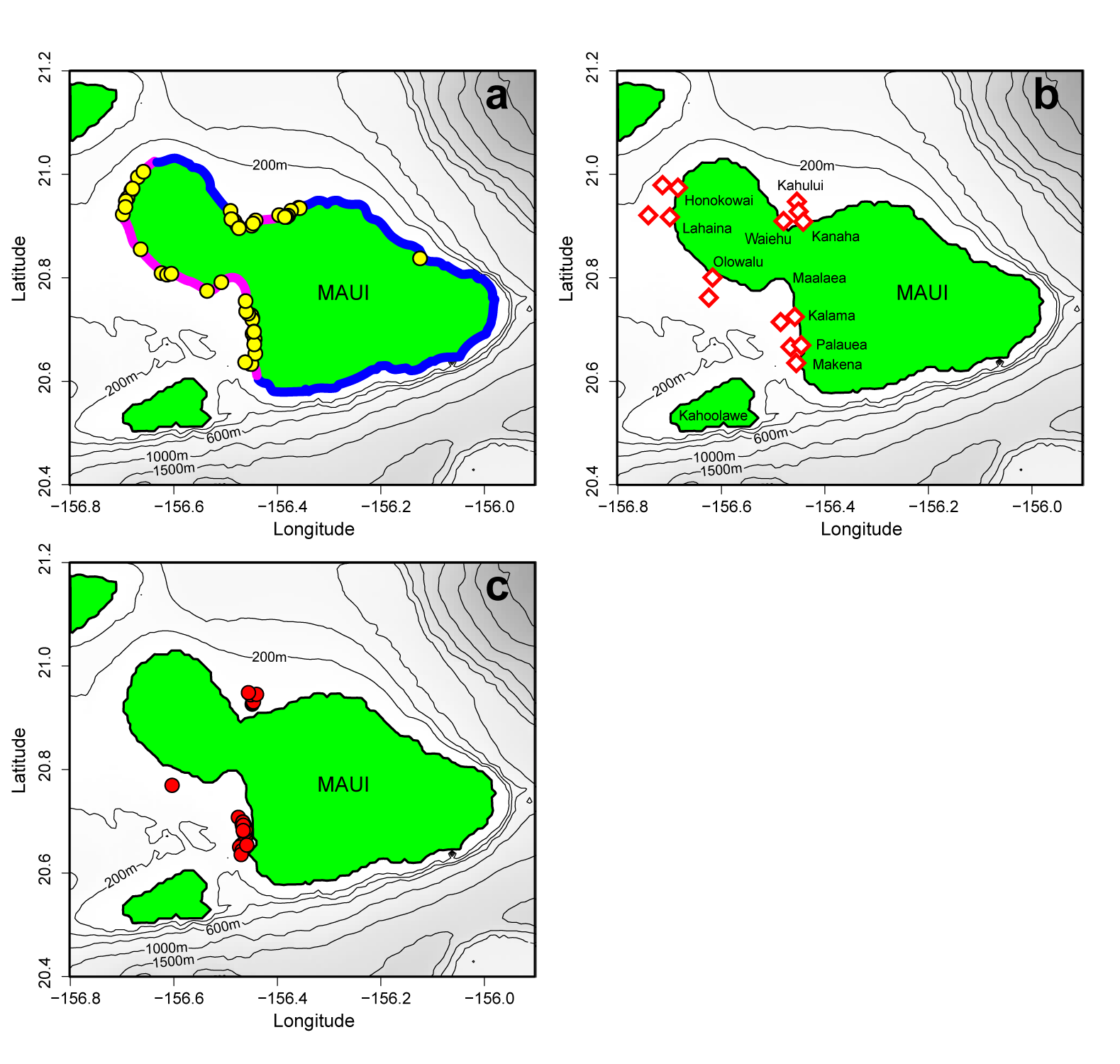


Supplementary Figure S14. Maui island showing locations of (a) shark bite incidents from 1980 to 2015 along high recreational-use (pink) and low recreational-use (blue) coastlines, (b) acoustic receiver monitoring locations, and (c) tiger shark tagging locations in 2013 and 2014. Maps were created using R software v.3.1.2 (R: A Language and Environment for Statistical Computing, R Core Team, R Foundation for Statistical Computing, Vienna, Austria (2014) https://www.R-project.org).


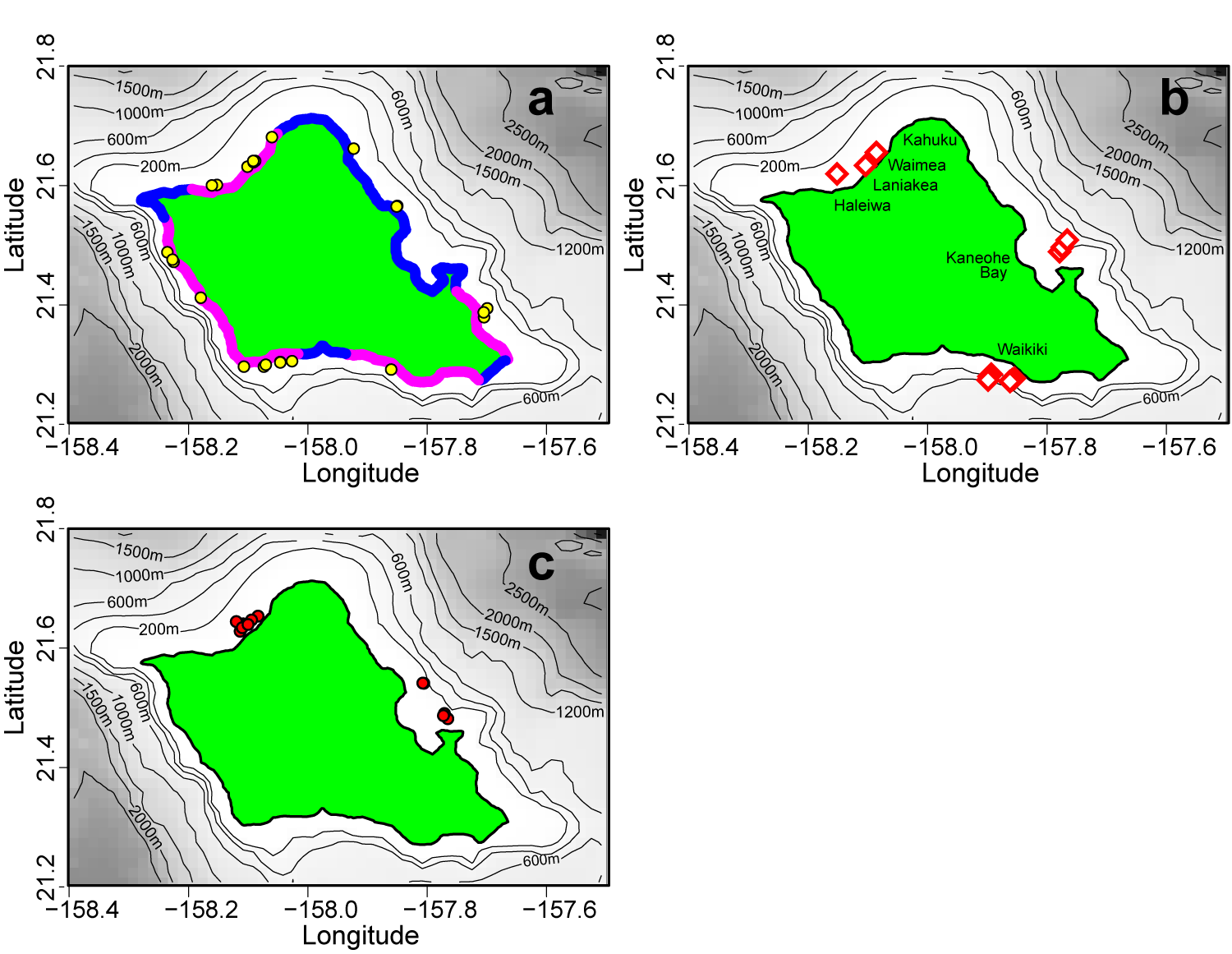


Supplementary Figure S15. Oahu island showing (a) locations of shark bite incidents from 1980 to 2015 along high recreational-use (pink) and low recreational-use (blue) coastlines, (b) acoustic receiver monitoring locations, and (c) tiger shark tagging locations in 2013 and 2014. Maps were created using R software v.3.1.2 (R: A Language and Environment for Statistical Computing, R Core Team, R Foundation for Statistical Computing, Vienna, Austria (2014) https://www.R-project.org).

**
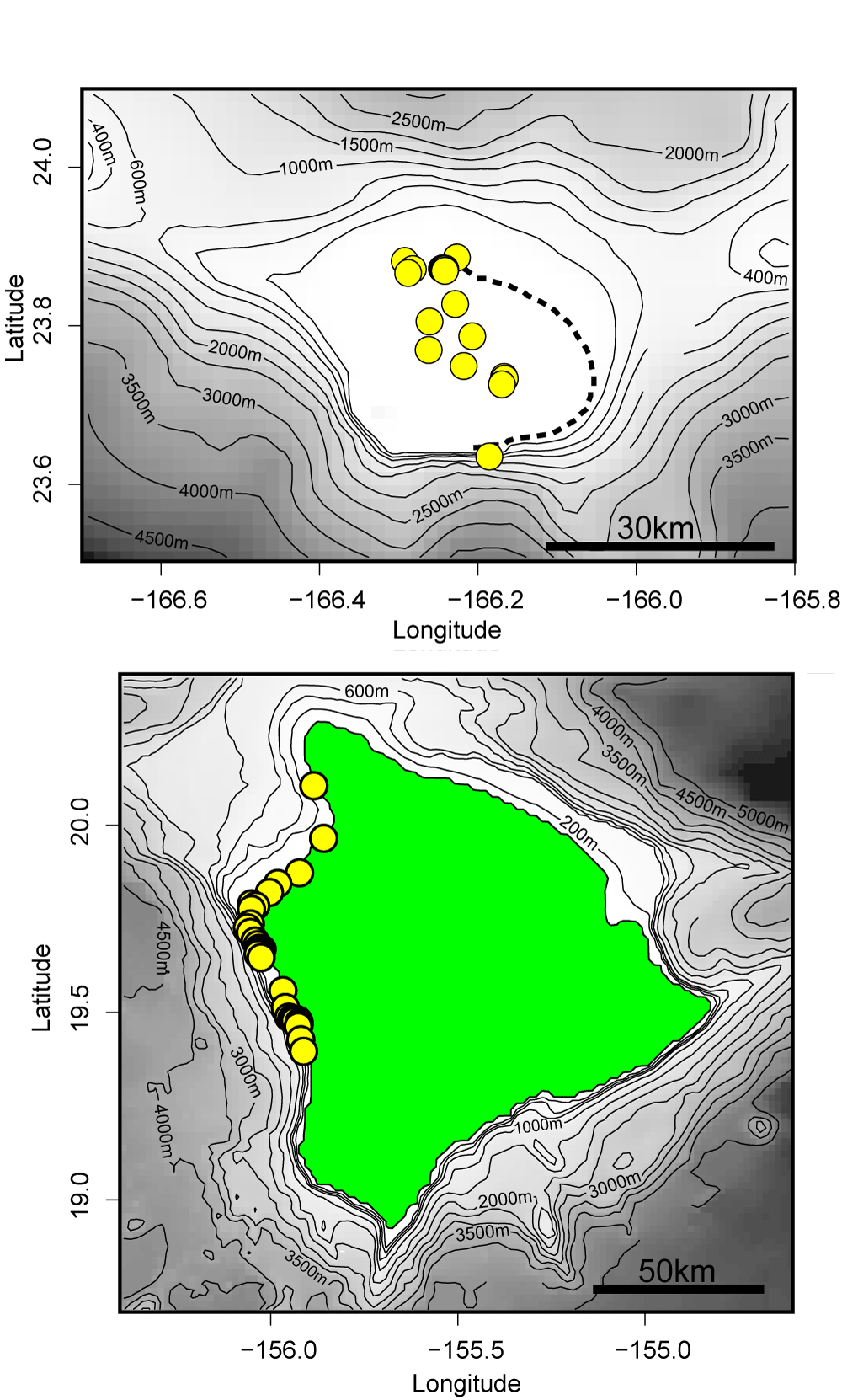
**

Supplementary Fig. S16. Receiver deployments (yellow points) at French Frigate Shoals (Top, dashed line indicates emergent barrier reef) and Hawaii Island (Bottom). Maps were created using R software v.3.1.2 (R: A Language and Environment for Statistical Computing, R Core Team, R Foundation for Statistical Computing, Vienna, Austria (2014) https://www.R-project.org).
